# Both EZH2 and JMJD6 regulate cell cycle genes in breast cancer

**DOI:** 10.1186/s12885-020-07531-8

**Published:** 2020-11-27

**Authors:** Antara Biswas, Geetashree Mukherjee, Paturu Kondaiah, Kartiki V. Desai

**Affiliations:** 1 National Institute of Biomedical Genomics, Kalyani, 741251 India; 2 Current address: Rutgers Cancer Institute of New Jersey, New Brunswick, NJ 08901 USA; 3 Kidwai Memorial Institute of Oncology, Bengaluru, 560029 India; 4 Current address: Tata Medical Center, 14 Main Arterial Road (EW), New Town, Rajarhat, Kolkata, 700156 India; 5 Molecular Reproduction, Development and Genetics, Indian Institute of Science, Bengaluru, 560012 India

**Keywords:** DREAM, Microarray, Prognosis, TNBC

## Abstract

**Background:**

Strong evidences support the critical role of Jumonji domain containing 6 (JMJD6) in progression of breast cancer. Here we explore potential partners that coregulate gene expression, to understand additional pathways that are activated by higher amounts of JMJD6.

**Methods:**

We used Gene Set Enrichment Analysis (GSEA) data to identify factors that display gene expression similar to cells treated with JMJD6 siRNA. Using chromatin immunoprecipitations (ChIP) against genomic regions that bind JMJD6 identified by in house and public database Encyclopaedia of DNA Elements (ENCODE), we confirmed JMJD6 occupancy by ChIP PCR. We tested the association of co-regulated genes with patient prognosis using The Cancer Genome Atlas (TCGA) datasets.

**Results:**

JMJD6 profiles overlapped with those of Enhancer of Zeste homolog 2 (EZH2) and together they appear to co-regulate a unique cassette of genes in both ER+ and ER- cells. 496 genes including aurora kinases, which are currently being tested as novel therapeutic targets in breast cancer were co-regulated in MDA MB 231 cells. JMJD6 and EZH2 neither inter-regulated nor physically interacted with one another. Since both proteins are chromatin modulators, we performed ChIP linked PCR analysis and show that JMJD6 bound in the neighbourhood of co-regulated genes, though EZH2 data did not show any peaks within 100 kb of these sites. Alignment of binding site sequences suggested that atleast two types of binding partners could offer their DNA binding properties to enrich JMJD6 at regulatory sites. In clinical samples, JMJD6 and EZH2 expression significantly correlated in both normal and tumor samples, however the strongest correlation was observed in triple-negative breast cancer (TNBC) subtype. Co-expression of JMJD6 and EZH2 imposed poorer prognosis in breast cancer.

**Conclusions:**

JMJD6 and EZH2 regulate the same crucial cell cycle regulatory and therapeutic targets but their mechanisms appear to be independent of each other. Blocking of a single molecule may not axe cell proliferation completely and blocking both JMJD6 and EZH2 simultaneously may be more effective in breast cancer patients.

**Supplementary information:**

**Supplementary information** accompanies this paper at 10.1186/s12885-020-07531-8.

## Background

Earlier we identified *JMJD6* as a gene that associates with poor prognosis in women with breast cancer [1]. We showed that *JMJD6* promoted cell proliferation and motility in breast cancer cell lines. *JMJD6* invoked increased expression of cell cycle genes such as Cyclin dependent kinase E2 (CCNE2) and simultaneously negated the expression of the classical cell cycle inhibitory pathway controlled by the transforming growth factor beta family ligands and receptors [1]. JMJD6 demethylates Estrogen receptor (ER), regulates histone H2A.X phosphorylation, cooperates with c-Myc in mouse mammary tumors and hydroxylates the tumor suppressor p53 protein supporting tumor growth and invasiveness [2–5]. JMJD6 was shown to regulate transcription via hydroxylation of Lysine 5 on histones [6]. Though JMJD6 has no known direct DNA binding activity, it has major impact on overall transcription. It releases stalled RNA polymerase from poised transcription start sites in the presence of Bromodomain containing protein 4 (BRD4) [7]. To define JMJD6 regulated transcriptional activity in breast cancer, we perturbed its expression in breast cancer cells and catalogued the expression levels of genes pertinent to poor prognosis and metastasis in breast cancer patients. We identified HOX transcript antisense intergenic RNA (*HOTAIR*), a long intervening non-coding RNA that is associated with poor survival and metastasis induced death in breast cancer patients as a transcriptional target of JMJD6 [8]. We identified a 21 base pair (bp) region that bound JMJD6 protein within 216 bp upstream of the transcriptional start site (TSS) of *HOTAIR* [8]. Our data also showed that when JMJD6 and HOTAIR are co-expressed in the same patients, patient survival is lower than in patients with expression of either one of the two genes [8].

When we queried the Gene Set Enrichment Analysis (GSEA) database for profiles that matched JMJD6 regulated gene expression cassettes, EZH2 mediated gene regulation was the most significant profile to be identified. Enhancer of Zeste 2 (EZH2) protein is a histone methylase and one of the constituent proteins of PRC2 that directly binds *HOTAIR* and determines its genomic location and distribution. During normal development, *HOTAIR* is known to guide the Polycomb repressive complex 2 (PRC2) to target sites like the HOX D cluster, enhancing trimethylation of Histone H3 at lysine 27 (H3K27me3) and silencing these genes [9]. In cancer, EZH2 was shown to redirect *HOTAIR*, to metastasis suppressor protocadherin genes, *PCHD5* and *PCHD10* and increase the H3K27me3 mark to silence gene expression. Loss of metastasis suppressor genes led to increased cell invasion and metastasis in breast cancer cells [10]. Since we had previously identified HOTAIR as a target of JMJD6, this established a possible link between JMJD6 and EZH2. Curiously, JMJD6 enhanced motility but was incapable of inducing metastasis in breast cancer cells [1]. In addition, real-time PCR data showed that PCHD5 and PCHD10 were positively regulated by JMJD6 (personal observations) and JMJD6 binding is associated with the activation mark H3K27Ac as opposed to H3K27me3 [11].

On the other hand, EZH2 also acts independently of HOTAIR and PRC2. Via a non-canonical pathway, EZH2 methylates and activates STAT3 (Signal Transducer And Activator Of Transcription 3), acts as a co-activator for androgen receptor (AR) in castration resistant prostate cancer and enhances cellular proliferation via the E2F pathway (reviewed in [12]). This established a second link between EZH2 and JMJD6, as both appear to induce cell proliferation. Therefore, both EZH2 and JMJD6 proteins are epigenetic regulators, histone modifiers and transcription factors and associate with poorer survival in breast cancer patients [1, 13]. Together, these evidences encouraged us to explore possible functional links between them and their impact on breast cancer progression.

In this manuscript we investigate both the physical and functional relationship between *JMJD6* and *EZH2*. We have used in silico data guided experimental approaches utilizing published ChIP-seq profiles and microarray experiments involving the two proteins. Our results show that JMJD6 and EZH2 regulate multiple genes that are both common and unique to each cell line. We could demonstrate direct binding of JMJD6 to the regulatory regions of multiple cell cycle genes that were commonly regulated in both these cell lines. In contrast, EZH2 showed no binding sites within 100 kb of these genes in in silico data and possibly controls cell cycle gene expression by using alternate mechanisms.

## Methods

### Cell culture

MCF-7 (HTB-22), MDA MB 231 (HTB-26) and HEK 293 (CRL-1573) cells were obtained from American Type Culture Collection (ATCC, Virginia, USA). The cell lines were cultured in Dulbecco’s modified Eagle’s medium (Gibco, USA) with 5% fetal bovine serum (Gibco, USA) and 1% Penstrep (Gibco, USA) in humidified 5% CO_2_ incubator at 37 °C. All cell lines were negative for mycoplasma presence (Lonza, Switzerland).

### Perturbation of gene expression levels

Construction of wild type JMJD6-V5 and generating stable clones of this construct in MCF-7 cells (J1-C), parental empty vector transfected control (Vec) and siRNA mediated knockdown of *JMJD6* has been described earlier [1, 8]. siRNAs with differential efficacies in knockdowns were used as follows - *JMJD6* siRNAs: siRNA A (Ambion: 111915) – 5′-GCUAUGGUGAACACCCUAATT-3′, siRNA C (Dharmacon: D-010363-02) – 5′-GGAUAACGAUGGCUACUCA-3′; *EZH2* siRNAs - siRNA E (Ambion: s4916) – 5′- GCUGACCAUUGGGACAGUATT-3′, siRNA F (Ambion: s4918) – 5′- GGCACUUACUAUGACAAUUTT-3′. A non-targeting scrambled siRNA (siRNA Scr) served as a negative control (Ambion: 4635). Transfections were carried out using Lipofectamine 2000 (Life Technologies, USA) using standard protocols for cell lines.

### RNA isolation, reverse transcription and quantitative real-time PCR

Total RNA from cell lines and tissue homogenates were isolated using Trizol (Invitrogen, CA) as per the manufacturer’s instructions described earlier [8]. Briefly, 1 μg of RNA was reverse transcribed and 1/10th of its cDNA was used per real-time PCR assay using SYBR green mix (Thermo Fisher Scientific, USA). C_T_ values using gene specific primers were normalized to the C_T_ value of *Actin* for cell lines and TATA-Box Binding Protein (*TBP*) for clinical samples since the latter showed the least variable expression in tumor and normal tissues. Gene expression values of vector alone were set to unity and fold change was calculated by 2^-eΔΔC^_T_ for cell lines. In case of clinical samples, for each gene median expression C_T_ value was calculated for normal samples. On this median, normal and tumor sets C_T_ values were normalized to calculate ΔΔC_T_ and Log_2_ transformed to calculate Log_2_ fold change. Primers used are listed in Additional file 1.

### Western blots

Cells were lysed in HNET buffer (25 mM HEPES-NaOH, 150 mM NaCl, 5 mM EDTA, 1% Triton X-100, 1 mM DTT, 1X Protease Inhibitor Cocktail (P2714, Sigma-Aldrich)), quantified by bicinchoninic acid (BCA) method (Pierce, USA) and equal amounts (50 μg or total cell lysate) were analyzed on 10–12% SDS-PAGE gels. Proteins were transferred for immunoblot analysis by standard methods. Primary antibodies were incubated at 4 °C overnight and suitable HRP conjugated secondary antibodies for 1 h at room temperature. Signals were detected with enhanced chemiluminescence (ECL) reagent (Pierce, USA) using Chemidoc (Bio-Rad, CA, USA). Antibodies used for western blots were JMJD6 for total protein (endogenous and exogenous) (PSR H-7, sc-28,348, Santa Cruz, CA, USA; 1:250), V5 for only exogenously expressed V5-tagged JMJD6 (R960–25, Invitrogen; 1:1000), EZH2 (AC22, Cell Signaling, MA, USA; 1:1000) and actin beta (A2228, Sigma, Missouri, USA; 1:500).

### Chromatin Immunoprecipitation and PCR

Chips were performed as described in [8]. Briefly, ChIP material was reverse cross-linked at 65 °C for 4 h, purified using PCR purification kit (Invitrogen, USA) and used for enrichment analysis using primers listed in Additional file 1. Antibodies used were same as for westerns except for EZH2 (ab3748, Abcam).

### ENCODE datasets

We downloaded publically available ENCODE ChIP-seq data for EZH2 in MCF-7 cells (accession number ENCSR906IQU). We retained the original author’s existing peak calls. EZH2 occupancy was determined for the promoter sites of the subset of genes having JMJD6 occupancy on promoter.

### Clinical samples

Breast cancer tumor samples and normal breast tissues were obtained from women who underwent surgery at Kidwai Memorial Institute of Oncology, Bangalore. Prior informed consent from patients was taken and the study was approved by the Institute Ethical Committee of Kidwai Memorial Institute of Oncology. RNA from 63 tumors and 23 normal samples was used for reverse transcription quantitative PCR and analyzed essentially as described by Damineni et al [14]. Pearson and Spearman Rank correlation tests and linear regression analysis was carried out between the genes and the best fit line was plotted using GraphPad Prism 7 software.

### Correlation and survival analysis

Gene expression values for *JMJD6* and *EZH2*, prediction analysis of microarray 50 (PAM50) classifications of tumors were merged with survival data obtained from TCGA breast cancer gene expression RNA-Seq data for 1108 tumors and 139 normal samples (http://xena.ucsc.edu/). Tumors lacking expression data, survival data or with duplicate representation were excluded. The median value of gene expression for *JMJD6*, and *EZH2* was calculated to group patients with above median value as high expressers and below median value as low expressers. Survival analysis to generate Kaplan–Meier curves using Log-rank statistics was carried out and plotted using GraphPad Prism 7 software. Data for *JMJD6* and *EZH2* protein and RNA expression from 70 and 960 samples respectively were obtained from cBioportal (https://www.cbioportal.org) to perform correlation and regression analysis and generate graphs.

### Statistical analysis

At least three independent experiments were performed for each assay. Statistical significance between different groups was determined using Student’s t-test and considered significantly different at *p* ≤ 0.05. The data was plotted using GraphPad Prism 7.0 software.

## Results

### Comparison of genes regulated by *JMJD6* and *EZH2*

**Fig. 1:**
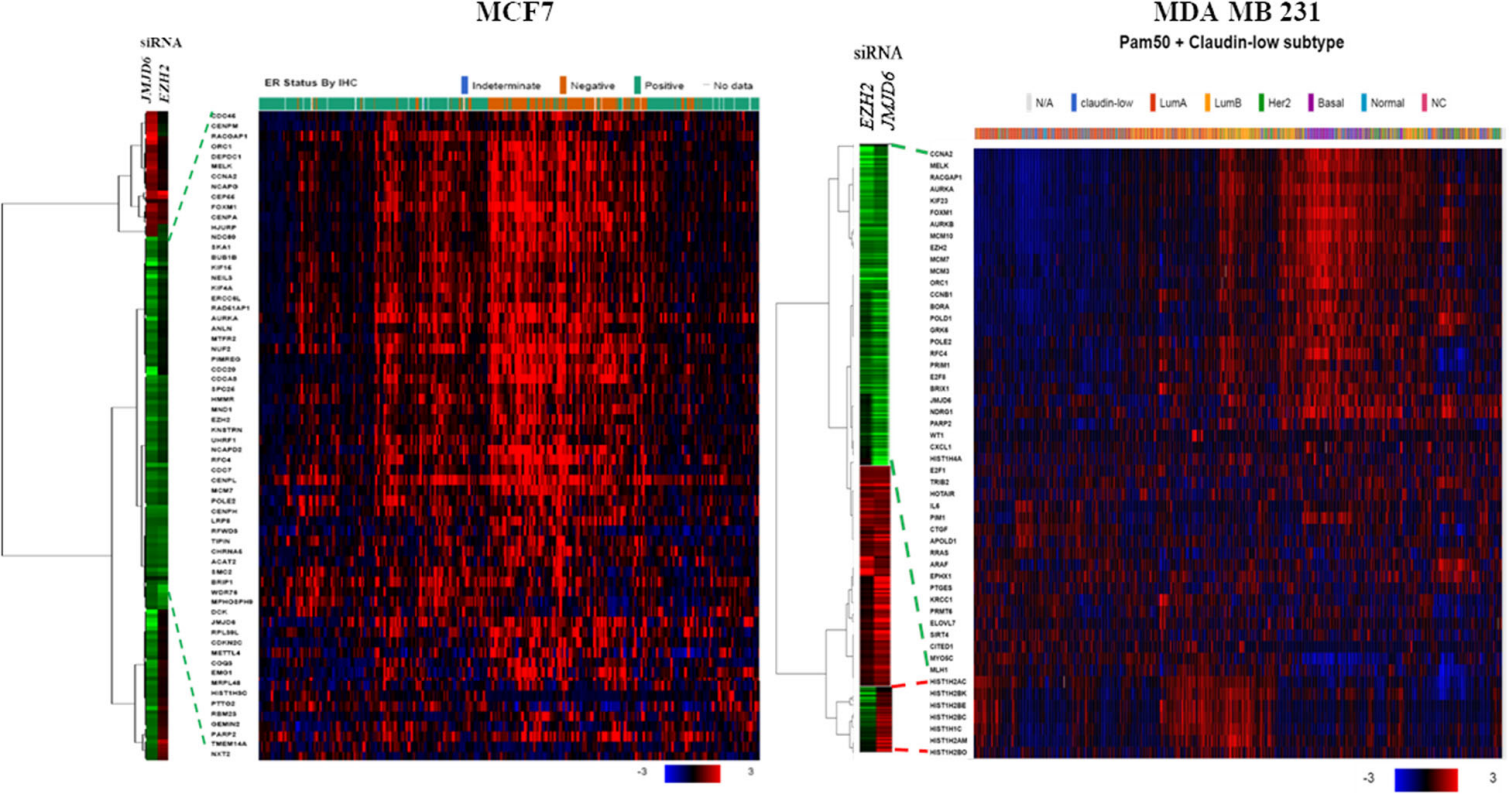
Expression Pattern of genes commonly regulated by *JMJD6* and *EZH2* siRNAs in TCGA breast cancer dataset. Expression levels of the representative genes (Z-scores) identified by expression array in MDA MB 231 cells (*n* = 496 genes) and MCF7 (*n* = 155) are represented. Red symbolizes up-regulated and green down-regulated genes. Hierarchical clustering was performed using CLuster and Treeview software

Microarray profiles using *JMJD6* overexpression and siRNA mediated depletion in MCF-7 and MDA MB 231 cells have been generated by us previously (accession number GSE31782). Comparison of these profiles using GSEA identified that *JMJD6* profiles overlapped with those regulated by *EZH2,* and its known targets *NIPP1* and *BMI1* (Table 1). The data for *EZH2* siRNA treated breast cancer cells was publicly available and downloaded for analysis (accession number GSE30670) [15]. To identify co-regulated genes, microarray data from MCF-7 and MDA MB 231 cells treated with siRNAs for *JMJD6* and *EZH2* were analyzed separately. EZH2 participates in constitutive activation of Nuclear factor kappa B (NFκB) regulated gene expression only in estrogen receptor (ER) negative MDA MB 231 but silences its expression in ER+ MCF-7 cells. EZH2 activity is therefore highly context specific in ER+ versus ER- cells [15] . Because of this regulatory dichotomy shown by EZH2, we chose to assess the combined activities of JMJD6 and EZH2, in both these cell lines as model systems. The overlapping gene lists can be found in Additional files 2 and 3. 155 and 496 genes were similarly regulated in MCF-7 and MDA MB 231 cells respectively (Fig. 1). GSEA analysis of both gene lists was performed and results show that dimerization partner (DP), RB-like, E2F and MuvB (DREAM) targets were enriched in both cell lines (Additional file 4). Out of the co-regulated DREAM target genes, majority were found to be regulated in a common direction in both cell-lines tested and these genes are listed in Additional file 5. In MDA MB 231 cells, we found that genes in the E2F pathway (29 genes), DREAM targets (112 genes), DNA replication and repair genes were induced by both *JMJD6* and *EZH2* whereas the expression of tumor suppressor genes was decreased. Genes regulated in opposite direction in MCF-7 cells did not fall into a unique pathway whereas histones were the only genes suppressed by *JMJD6* but induced by *EZH2* in MDA MB 231 (Fig. 1).

**Table 1:** GSEA analysis of JMJD6 regulated genes

Gene Set Name	# Genes in Gene Set (K)	Description	# Genes in Overlap (k)	k/K	***p*** value
NUYTTEN_NIPP1_TARGETS_DN	777	Genes down-regulated in PC3 cells (prostate cancer) after knockdown of NIPP1 [Gene ID = 5511] by RNAi.	97	0.1248	0.00E+ 00
NUYTTEN_EZH2_TARGETS_UP	974	Genes up-regulated in PC3 cells (prostate cancer) after knockdown of EZH2 [Gene ID = 2146] by RNAi.	123	0.1263	0.00E+ 00
NUYTTEN_EZH2_TARGETS_UP	974	Genes up-regulated in PC3 cells (prostate cancer) after knockdown of EZH2 [Gene ID = 2146] by RNAi.	64	0.0657	1.31E-10
HAN_SATB1_TARGETS_DN	331	Genes down-regulated in MDA-MB-231 cells (breast cancer) after knockdown of SATB1 [Gene ID = 6304] by RNAi.	57	0.1722	0.00E+ 00
DOUGLAS_BMI1_TARGETS_UP	512	Genes up-regulated in A4573 cells (Ewing’s sarcoma, ESFT) after knockdown of BMI1 [Gene ID = 648] by RNAi.	103	0.2012	0.00E+ 00
ONDER_CDH1_TARGETS_2_UP	257	Genes up-regulated in HMLE cells (immortalized nontransformed mammary epithelium) after E-cadhedrin (CDH1) [Gene ID = 999] knockdown by RNAi.	45	0.1751	2.44E-15

To test if these changes in gene expression were reflected in the human breast cancer RNA-seq data, the TCGA datasets were queried for the expression of both MCF-7 (155 genes) and the 496 MDA MB 231 gene sets to determine if the cell line effects of *JMJD6* and *EZH2* could be observed in patient samples. As shown in Fig. 1, the MCF-7 155 set showed similar regulation in ER negative and about 50% of the ER+ tumors indicating that these genes were regulated by both JMJD6 and EZH2 commonly across ER+ and ER- tumors. Interestingly, these same tumors also showed higher expression of JMJD6 and EZH2 supporting the idea that these two could be possible regulators of these genes in human tumors too. Further, out of the MDA MB231 496 genes, most recapitulated the expression patterns found in the cell line MDA MB 231in the TNBC subtype. On closer examination, it was the DREAM and E2F targets that were commonly regulated in both ER+ and ER- tumors. Since a larger number of genes correlated with the TNBC subset, it is possible that *JMJD6-EZH2* axis may play a significant role beyond cell cycle regulation in TNBC tumor biology.

### Concurrent high expression of *JMJD6* and *EZH2* is associated with breast cancer progression and poor prognosis

**Fig. 2:**
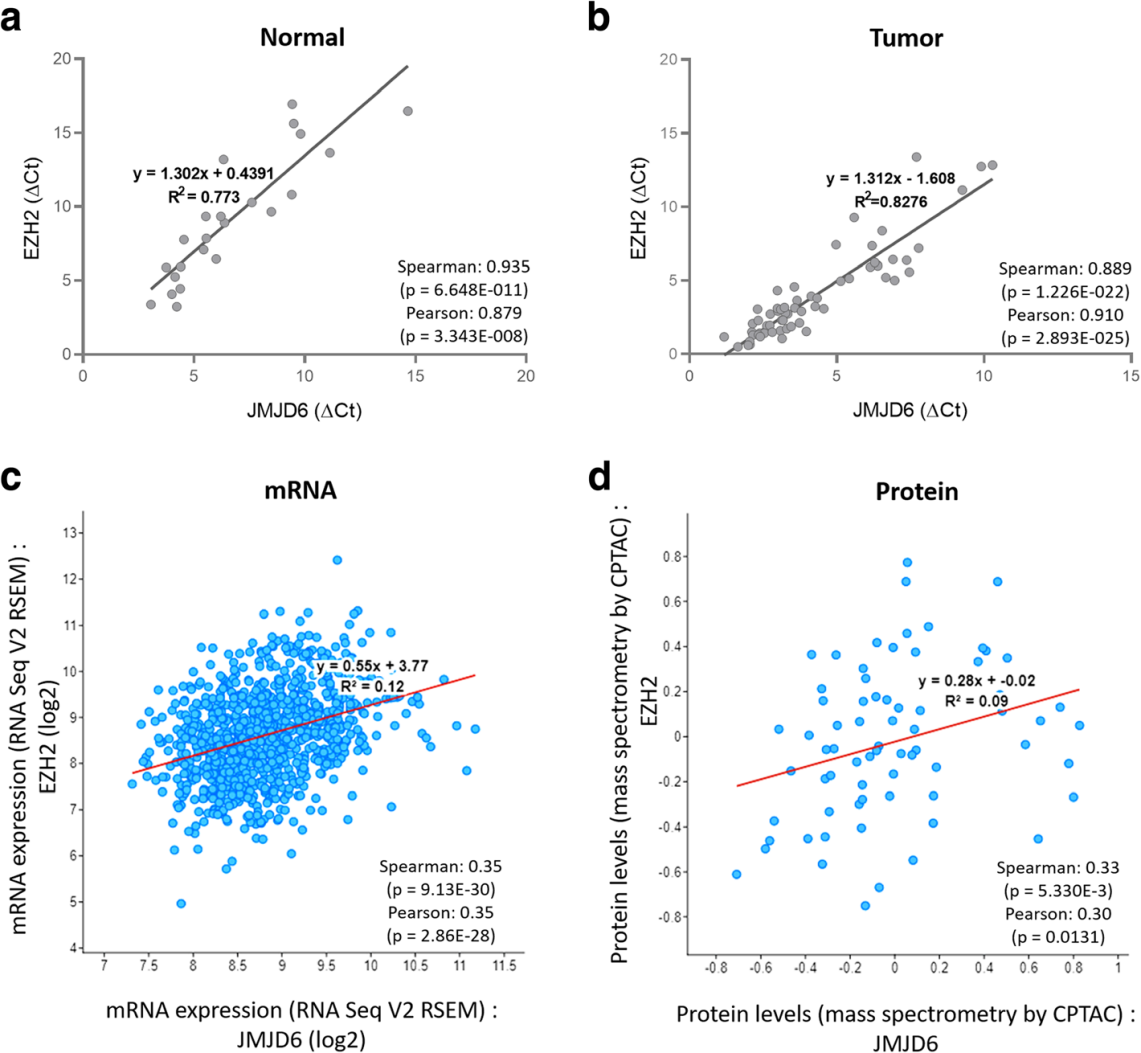
Linear regression analysis of the expression of *JMJD6* and *EZH2* in patient derived RNA. Graphs show the best fit regression line in Normal (**a**) and Tumor (**b**) samples for *JMJD6*-*EZH2* where X-axis and Y-axis show normalized expression (ΔCt) of respective genes and in *JMJD6* and *EZH2* altered samples from TCGA RNA-Seq and mass spectrometry data for mRNA (**c**) and protein (**d**) expression respectively

Since, JMJD6-EZH2 regulated overlapping genes in both ER+ and ER- cell lines; we investigated if they are co-expressed in 63 tumor samples and 23 adjacent/independent normal breast samples by real-time PCR analysis. Pearson and Spearman correlation tests revealed significant overall correlation between *JMJD6* and *EZH2* in both normal and tumor samples irrespective of the tumor histopathology (DCIS, IDC1 to IDC3) or molecular subtype (TNBC versus non-TNBC) (Table 2). Though the highest correlation co-efficient was found in the TNBC subtype (Table 2, Additional file 6), JMJD6 and EZH2 were co-expressed in all samples/cells studied. This suggests that correlation between *JMJD6*-*EZH2* expressions may not be tumor specific, although the correlation was better in tumor than in normal samples. Linear regression analysis also showed a strong and consistent correlation between *JMJD6* and *EZH2* expression (Fig. 2a, b). In silico analysis of publicly available RNA and protein expression data from TCGA datasets recapitulated cell line data and *JMJD6* and *EZH2* were positively correlated and co-expressed in breast cancer samples (Fig. 2c, d). Interestingly, Kaplan-Meier analysis using expression and survival data of breast cancer patients from TCGA (https://xena.ucsc.edu) showed that there was a trend towards a considerable shorter survival in patients with simultaneous upregulation of *JMJD6* and *EZH2* expression (Additional file 7).

**Table 2:** Correlation between *JMJD6* and *EZH2* expression in breast normal and tumor samples before and after subclassification based on histopathological and molecular subtyping

	***JMJD6-EZH2***			
# Samples (n)	Pearson (r)	***p***-value	Spearman (rs)	p-value
Normal (23)	0.879	3.00E-08	0.935	7.00E-11
Tumor (63)	0.91	6.00E-25	0.889	2.00E-22
**Type**				
IDC3 (42)	0.921	6.00E-18	0.829	1.00E-11
DCIS & IDC2(14)	0.916	4.00E-06	0.943	2.00E-06
**Subtypes**				
Non-TNBC (31)	0.864	4.00E-10	0.811	3.00E-08
TNBC (25)	0.965	8.00E-15	0.94	3.00E-12
ER+ (17)	0.877	4.00E-06	0.877	1.00E-05
ER- (39)	0.936	2.00E-18	0.858	3.00E-12
HER2+ (18)	0.806	5.00E-05	0.711	0.001
HER2- (38)	0.918	6.00E-16	0.92	4.00E-16

### JMJD6 and EZH2 do not bind each other nor inter-regulate one another

Since JMJD6 and EZH2 showed high correlation in expression patterns and showed overlapping gene expression patterns, and both *JMJD6* and *EZH2* are epigenetic regulators, bind regulatory regions, we hypothesized that they may interact with each other on a functional level [16, 17]. This interaction could be a result of a) Physical interaction between JMJD6 and EZH2 proteins, recruitment to common regulatory regions of target genes; b) these genes have a hierarchical regulatory relationship between one another. That is, one gene is the downstream target of the other (for example-JMJD6 regulates EZH2) and siRNA mediated knockdown of the regulator (JMJD6) results in loss of expression of the target gene (EZH2), in turn leading to similar and overlapping expression patterns in siRNA experiments.

First, co-immunoprecipitation assays were carried out to determine if JMJD6 and EZH2 physically interact with each other, but no such interaction was observed (data not shown). To explore any hierarchical regulatory relationship between the two genes we studied their expression after different perturbations in gene expressions. Since co-expression was evident in both ER+, ER- and non-cancerous samples, we used three corresponding representative cells to determine the relationship between JMJD6 and EZH2 expression. 1) Measure EZH2 expression in MCF7 ER+ cells that overexpressed JMJD6 (J1-C clones) 2) Measure the expression of EZH2 in JMJD6 siRNA treated, and JMJD6 levels in EZH2 siRNA treated MDA MB 231 cells 3) Measure expression in HEK293 cells, which represent a non-cancerous cell population. Both RNA and protein expression of JMJD6 and EZH2 was measured using real-time quantitative PCR and immunoblotting respectively.

**Fig. 3:**
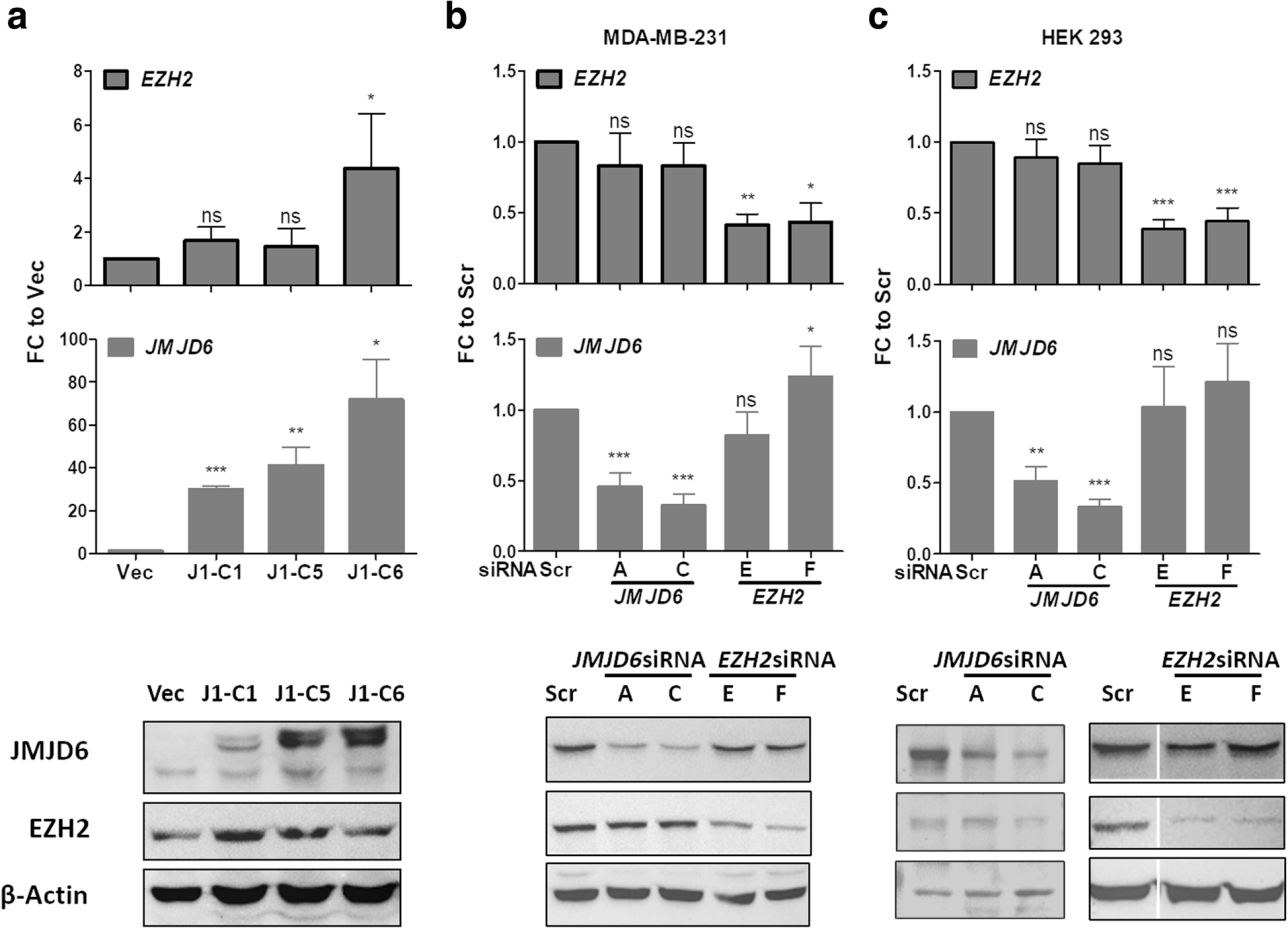
Inter-regulation of *JMJD6* and *EZH2*. **a**) Expression of *JMJD6* and *EZH2* in different JMJD6 overexpressing clones as quantified by real-time PCR, *JMJD6* and *EZH2* levels following *JMJD6* siRNAs and *EZH2* siRNAs is shown in **b**) for MDA MB 231 and **c**) for HEK 293 cells. Lower panels show JMJD6 and EZH2 proteins detected by immunoblot analysis. Endogenous JMJD6 is marked by filled squares and empty squares indicate V5-tagged protein (right hand side of the immunoblot panel). Actin beta (ACTB) was used as an internal control

No consistent and remarkable change in *EZH2* levels was observed (except a small change in J1-C6 clone in MCF-7 cells overexpressing JMJD6 (Fig. 3)a. As shown in Fig. 3(b and c), *JMJD6* siRNAs failed to deplete *EZH2* levels and similarly, *EZH2* siRNAs failed to decrease *JMJD6* levels in both MDA MB 231 and HEK 293 cells. These proteins, therefore, do not appear to inter-regulate each other’s expression at neither the RNA nor the protein level. Therefore, there does not appear to be any hierarchical gene regulatory relationship between them. We attempted co-treatment of cells using siRNA against both JMJD6 and EZH2, however, the cells failed to survive. We could not perform direct assays such as proliferation, motility, colony formation and invasiveness following removal of both genes to conclusively show that they act in unison to promote tumor growth or progression.

### JMJD6 binds regulatory regions in target genes

We further explored if *JMJD6* and *EZH2* bind near genes they co-regulate individually or by co-binding overlapping regions in the regulatory regions of target genes that were regulated in both MCF-7 and MDA MB 231 cells. We used publicly available EZH2 ChIP-Seq data in MCF-7 cells (accession number ENCSR906IQU) and previously published JMJD6 ChIP data in HEK 293 and HeLa cells [7]. For this method, we used a gene by gene approach and looked for potential peaks within 100 kb upstream and downstream of the transcription start site (TSS) of potential DREAM targets, genes validated for EZH2 binding previously and those that were implicated in patient prognosis or are being explored as potential targets in cancer that appeared in our gene expression list. We particularly found peaks for JMJD6 within 40 kb of the TSS of many regulated genes and used regional genomic co-ordinates by displaying the bigwig data files in the UCSC browser (data not shown). However, EZH2 rarely had peaks within the vicinity, though we cannot rule out the possibility that such potential binding sites may occur distal to the 100 kb region we scanned.

**Fig. 4:**
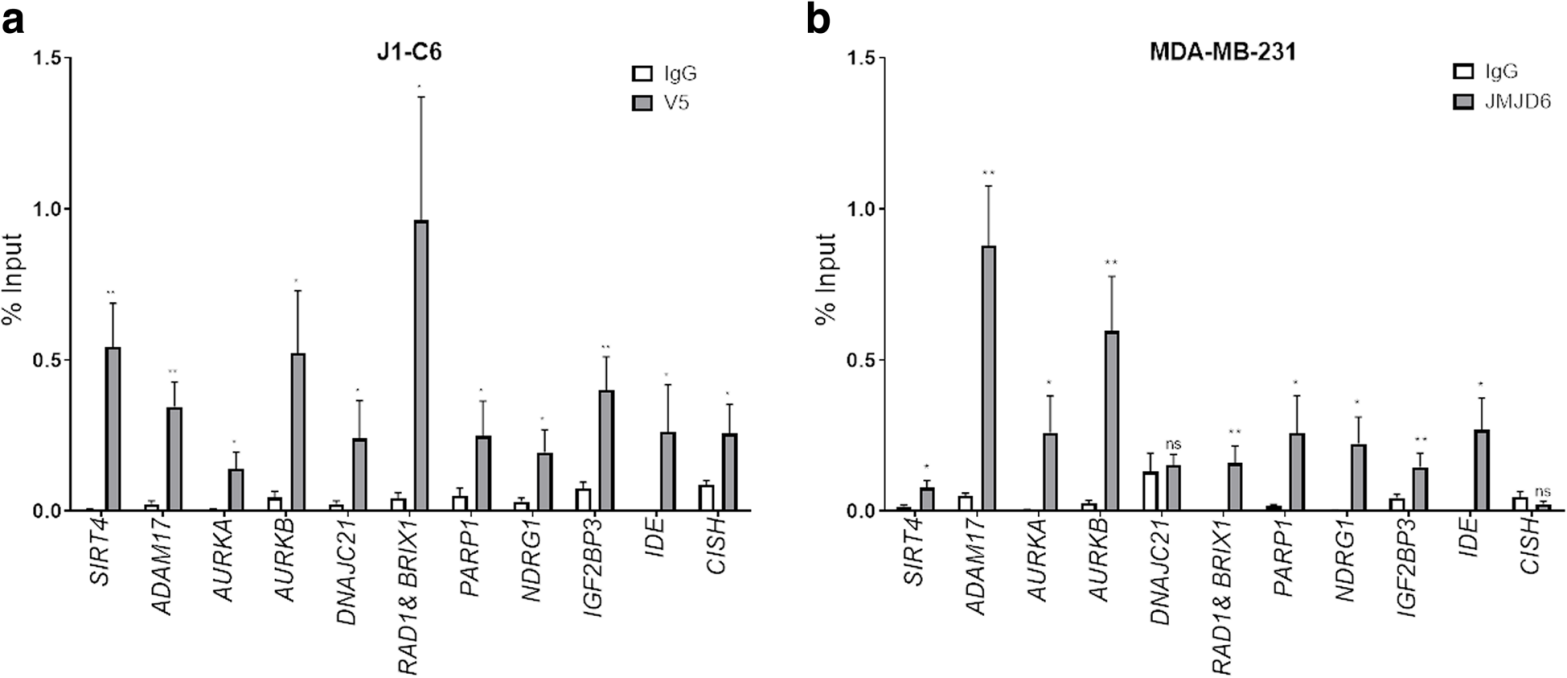
Recruitment of JMJD6 in the regulatory regions of co-regulated genes. Percentage input enrichment for ChIP is shown in **a**) J1-C6 cells using V5-antibody, **b**) MDA MB 231 cells using JMJD6 antibody for representative co-regulated genes

We chose few individual genes listed in Table 3 exhibiting binding by JMJD6 and made primers flanking the peak sequences. Since the peaks were derived from cells other than MCF-7 and MDA MB 231, we validated JMJD6 binding potential in these cells using V5-and JMJD6 ChIP experiments. Figure 4a shows that genomic regions for all the genes could be captured by real-time PCR and showed robust enrichment in J1-C6 cells. However, in MDA MB 231 cells most sites except DNAJC21 and CISH, showed good enrichment (Fig. 4b). To further characterize EZH2 binding, its binding profiles were checked from ENCODE in MCF-7 cells. EZH2 was found to have peaks with negligible enrichment for most sites except *PARP1* and *CISH* genes (data not shown), indicating JMJD6 and EZH2 may not co-bind regulatory regions of all genes they co-regulate.

**Table 3:** Peak regions associated with regulated genes used for PCR validation of JMJD6 binding in ChIP assays

Gene Symbol	Gene name	Chromosome	Chromosomal location of gene	Chromosomal location of peak	Peaks associated with /flanking genes	Distance from nearest TSS (kb)
*IGF2BP3*	Insulin Like Growth Factor 2 MRNA Binding Protein 3	7	23,349,828–23,510,086	23,553,064–23,553,529	Downstream	200
*SIRT4*	Sirtuin 4	12	120,740,119–120,751,052	120,734,229–120,734,659	Upstream	7
*AURKA*	Aurora Kinase A	20	54,944,445–54,967,393	54,982,773–54,983,281	Downstream	40
*AURKB*	Aurora Kinase B	17	8,108,049–8,113,918	8,066,787–8,067,363	Upstream	40
*ADAM17*	ADAM Metallopeptidase Domain 17	2	9,628,615–9,695,921	9,688,040–9,688,582	Downstream	60
*RAD1*	RAD1 Checkpoint DNA Exonuclease and	5	34,905,365–34,919,094	34,874,986–34,875,354	Upstream	30
*BRIX1*	BRX1, Biogenesis Of Ribosomes	5	34,915,481–34,926,101	34,874,986–34,875,354	Upstream	30
*DNAJC21*	DnaJ Heat Shock Protein Family (Hsp40) Member C21	5	34,929,698–34,959,069	34,951,185–34,951,607	Genic	–
*IDE*	Insulin Degrading Enzyme	10	94,211,441–94,333,852	94,323,996–94,324,470	Genic	–
*PARP1*	Poly (ADP-Ribose) Polymerase 1	1	226,548,392–226,595,801	226,667,173–226,667,562	Downstream	100
*NDRG1*	N-Myc Downstream Regulated 1	8	134,249,414–134,314,265	134,284,281–134,284,881	Genic	–
*CISH*	Cytokine Inducible SH2 Containing Protein	3	50,643,885–50,649,262	50,628,326–50,628,778	Upstream	15

### Characteristics of the JMJD6 binding regions

**Fig. 5:**
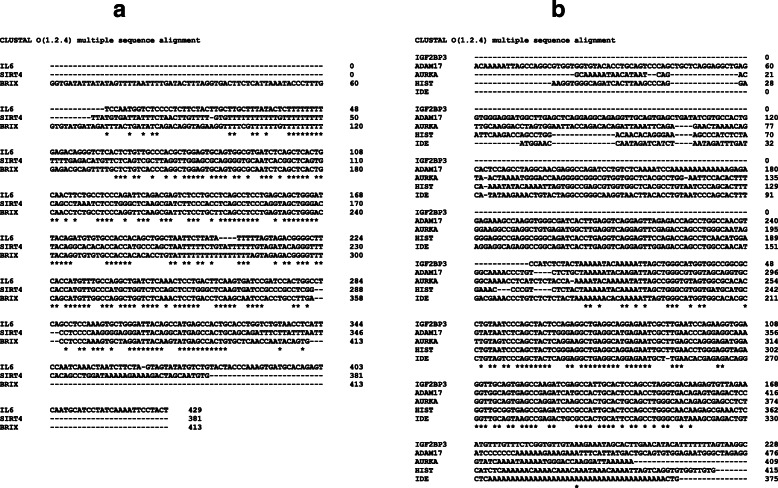
Multiple sequence alignment of binding sites of JMJD6. Two types of clusters are shown with **a**) *IL6*, *SIRT4*, *BRIX1*, and **b**) *IGF2BP3*, *ADAM17*, *AURKA*, Histones, *IDE* sites. The conserved sequences are marked by “*”

JMJD6 prefers binding ssRNA over DNA and since there is no overt DNA binding motif in JMJD6 protein, it possibly interacts with DNA via other genomic factors. To determine if the chosen and validated sites could point to a common potential factor(s), we aligned the 20 genomic locations in ClustalW using standard parameters and looked for conserved sequences. Interestingly the binding regions showed two types of clusters with *IGF2BP3, ADAM17, AURKA, IDE,* Histones forming one group and *IL6, SIRT4, BRIX1* sites forming another (Fig. 5). We used the conserved sequences to find out factors that could potentially bind these regions. These regions were enriched and densely packed for several transcription factor motifs (Additional file 8A and B). We compared this list of proteins with published JMJD6 mass spectrometry data and performed literature scans, however, we could not identify a factor that could be pursued for further studies. However, binding of JMJD6 has been established unequivocally. This data also suggest that regulation of DREAM targets may involve long distance cis-acting elements that are brought in context by JMJD6 binding to its sites.

## Discussion

In this paper, we studied *JMJD6*-*EZH2* functional interaction and found a subset of co-regulated genes by microarray analysis of *JMJD6* and *EZH2* siRNA treated cell lines. Individually, JMJD6 and EZH2 regulate 800 and 750 genes in MDA MB 231 cells. Together, they perturbed the expression of 496 genes suggesting that a high proportion (almost > 50%) of individually regulated genes were also regulated by both these factors. A portion of the 496 genes were positively regulated by both proteins, and these genes are known to be associated with aggressive behaviour in tumors. The down-regulated genes belonged largely to the tumor suppressors group of proteins (Fig. 1). Pathway analysis identified upregulated genes as targets of DREAM proteins, which are major cell cycle regulators (Additional file 4). DREAM is a transcriptional repressor complex which binds *E2F* and *CHR* promoter elements to repress G1/S and G2/M genes, regulating cell cycle-associated genes [18]. This suggests that JMJD6/EZH2 alleviates the repression enforced by DREAM proteins and probably induces E2F. We found 29 of E2F targets upregulated in our lists. Interestingly, in MCF-7 cells only 155 genes overlapped in the EZH2 and JMJD6 siRNA treated cells, most of the genes (*n* = 89) were DREAM targets. Cell cycle regulation including E2F action appears to be a common pathway regulated by both JMJD6 and EZH2, in both ER+ and ER- cells. This data is supported by Wong et al who recently showed that *JMJD6* downregulation significantly reduces the expression of E2F target genes in neuroblastoma cells [19]. Interestingly, EZH2 is a downstream target of E2F, has E2F binding sites in its promoter region and is required for E2F driven cell proliferation in both normal and cancer cells [20].

GSEA analysis of the overlapping genes from this study identified a dataset capable of sub-classifying ER+ patients with histologic grade 2 tumors into two groups based on high versus low risk of recurrence [21]. In genetic testing for risk of recurrence based on this study a panel of 196 individual genes are required to be tested by PCR methods. Since JMJD6 and EZH2 appear to regulate most of the 196 genes used in this test, assessing only these two genes for their expression values or their protein levels in tumor samples could be developed into a replacement assay for the 196 gene genetic test. How successfully are JMJD6 and EZH2 assays able to replace this genetic test in such sub-classification, needs to be assessed carefully in the future.

*JMJD6* and *EZH2* both are epigenetic modulators, removing and imparting methylation marks on histones, thus controlling expression of target genes. Since both regulate a unique subset of genes, their presence on regulatory regions of these genes was evaluated. Using a gene by gene approach, peaks near TSS of target genes from published JMJD6 and EZH2 ChIP-Seq data were selected. Out of selected genes, 12 representative genes were tested for binding, in particular the genes known to be *E2F*/*DREAM* targets and having prognostic value in breast cancer. Though most peaks chosen were negative for EZH2 binding, majority were positive for JMJD6 ChIP in both J1-C6 and MDA MB 231 cells (Fig. 4) indicating that EZH2 and JMJD6 were probably not co-binding cis-regulatory elements. The question remains if the two proteins were still capable of interacting at the protein-protein level and we were missing long distance interaction(s) on the regulatory sequences. In co-IP experiments we failed to detect an interaction between JMJD6 and EZH2. We also used JMJD6 antibody to pull down all interacting proteins in JMJD6 overexpressing MCF-7 cells and performed mass spectrophotometric analysis (unpublished data). Webby et al. published similar studies [22]. Both pull-downs did not find EZH2 as a potential JMJD6-bound protein. We confirmed this by co-immunoprecipitation assay using EZH2 antibody followed by immunoblotting for JMJD6 (data not shown). This indicates that though JMJD6 and EZH2 appeared to have an association with gene regulation they do not necessarily display direct protein-protein physical interaction. However, these experiments did not explore if these proteins can interact indirectly as a part of a larger protein complex.

Expression of these two genes showed a very high correlation in normal as well as tumor samples. Therefore, overlap in gene regulation by these two proteins could also be explained if EZH2 itself is regulated by JMJD6, probably via E2F [19, 20]. Or JMJD6 is regulated by EZH2 by as yet unknown mechanisms. Using siRNA depletion (*JMJD6*), we assessed RNA or protein level changes in the other gene (*EZH2*). As shown in Fig. 3, neither JMJD6 regulated EZH2 expression nor was the reverse true. Our data therefore indicates that though same set of genes are regulated, the action of JMJD6 and EZH2 appears to be independent of each other.

Since both *JMJD6* and *EZH2* associate independently with poor prognosis in cancer, the results were extrapolated to clinical samples, and their expression and association was studied in tumor and normal samples; and in publicly available RNA-Seq databases [1, 23–27]. *JMJD6* and *EZH2* appeared to be robust indicators of each other’s expression in both tumors as well as normal breast samples since they show very strong statistically significant correlation co-efficient in both samples (Fig. 2). Since these genes are probably always co-expressed, we used publicly available TCGA database to study the association between patient prognosis and their expression levels. Survival analysis of breast cancer patients up to 4 years (about 1500 days) showed poorer prognosis when expression of both *JMJD6* and *EZH2* was high, however it did not achieve statistical significance by the end of 5 years (Additional file 7). Clinical outcomes suggest that though high levels of these genes individually leads to poor prognosis, combined high expression made matters worse in patients. In TCGA dataset, JMJD6-EZH2 regulated genes were found to have higher expression in ER- samples than ER+ tumors (Fig. 1, MDA MB 231). TCGA data would suggest that these genes maybe more relevant in the TNBC subtype. Further, in the 155 genes identified in MCF-7 ER+ cells, 40 overlap with the DREAM targets that promote cell cycle entry and these genes were highly expressed in most ER- and about 50% of the ER+ samples in TCGA. These same 50% ER+ tumors had higher levels of EZH2 and JMJD6 expression. This observation supports the idea that these similarly regulated genes are probably also regulated by JMJD6 and EZH2 in ER+ as well as ER- cancer samples.

## Conclusions

**Fig. 6:**
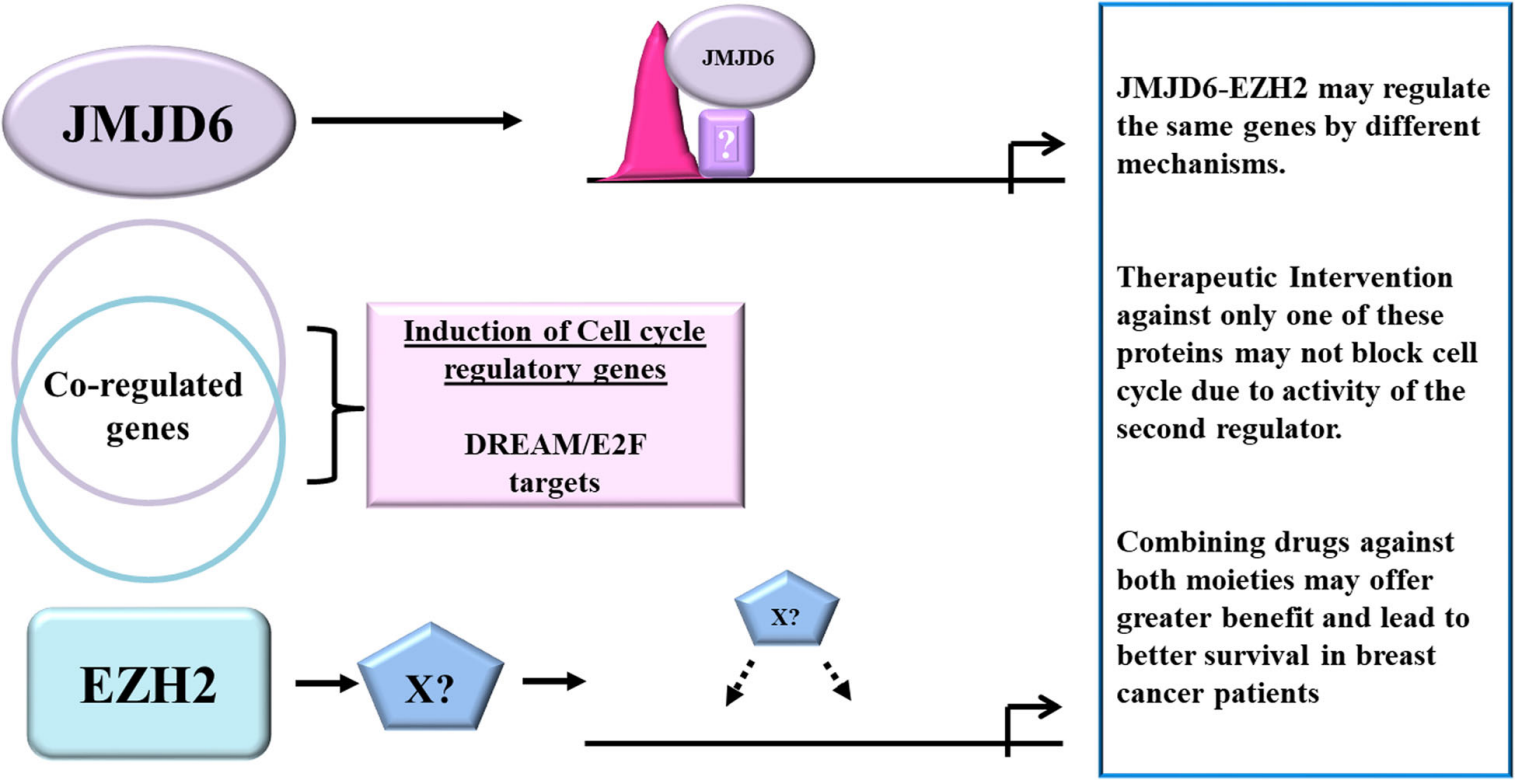
Graphical representation of JMJD6 and EZH2 action

In summary, this study identified a subset of genes regulated by *JMJD6* and *EZH2*, majority of which are cell cycle regulators. When combined together, *JMJD6* and *EZH2* appear to be better markers for poor prognosis, they function in both receptor positive/negative tumors and their expression levels may foretell the level of 196 genes that are used to sub-classify grade 2 ER+ tumors based on the risk of recurrence. However, no evidence was found to suggest that EZH2 and JMJD6 regulate each other’s expression and/or directly and physically bind each other. Though the visible outcome of perturbing either of the genes suggests co-regulation of downstream genes, the two proteins appear to use alternate mechanisms of regulating the same genes (Fig. 6). JMJD6 bound regulatory regions but EZH2 did not. Pharmacological intervention against JMJD6 would likely achieve a similar result to that of EZH2 inhibitors. If the regulatory mechanisms adopted by the two genes are independent of one another, the intervention against a single protein may not achieve complete shutdown of the cell cycle pathways and hence the desired therapeutic effect. A combinatorial therapy using drugs developed against both proteins may be a better strategy for complete shut-down of cell cycle. Both ER- and ER+ subtypes could benefit from this and improve survival of all patients suffering from breast cancer.

## Supplementary information

**Additional file 1.** List of primers used for real-time PCR and ChIP-qPCR.

**Additional file 2.** List of genes co-regulated by both *JMJD6* and *EZH2* in MCF-7 cells. FC represents Fold Change.

**Additional file 3.** List of genes co-regulated by both *JMJD6* and *EZH2* in MDA MB 231 cells. FC represents Fold Change.

**Additional file 4.** Gene Set Enrichment Analysis (GSEA) and the top 20 pathways found in 496 genes.

**Additional file 5.** DREAM target genes co-regulated by both *JMJD6* and *EZH2 (DOCX 14 kb)*.

**Additional file 6.** Sample wise expression levels of *JMJD6* and *EZH2* in the PCR analysis of 23 normal and 63 tumor samples. The X-axis show tumors arranged by increasing expression values of *JMJD6* in a breast cancer sub-type/group-wise manner (lower panel of the graph). Corresponding levels of *EZH2* are shown in the upper panel.

**Additional file 7.** Survival analysis based on *JMJD6* and *EZH2* expression*.* Kaplan Meier curves of overall survival for 5 years for breast cancer patients stratified by median into low and high expressers of *EZH2* (A) and combination of JMJD6 and EZH2 (B).

**Additional file 8.** Potential transcription factor binding sites in the conserved JMJD6 binding sites in two clusters types. Cluster with genes *IL6*, *SIRT4*, *BRIX1* (A), and *IGF2BP3*, *ADAM17*, *AURKA*, Histones, *IDE* sites (B). The sequence used for PATCH begins with first base of conserved sequence marked in the clusters and PATCH score is given as percent binding.

**Additional file 9.** Original full images of western blots used for generating composite Fig. 3.

## Data Availability

The datasets supporting the findings of this study are available in cBioportal (https://www.cbioportal.org/), ENCODE (https://www.encodeproject.org/) and TCGA repository (https://www.cancer.gov/about-nci/organization/ccg/research/structural-genomics/tcga).

## Abbreviations

ACTB: Actin beta
ChIP: Chromatin Immunoprecipitation
DCIS: Ductal carcinoma in situ
DREAM: dimerization partner (DP), RB-like, E2F and MuvB
ENCODE: Encyclopedia of DNA Elements
ER: Estrogen receptor
EZH2: Enhancer of Zeste homolog 2
GSEA: Gene Set Enrichment Analysis
HER2: Human epidermal growth factor receptor 2
HOTAIR: HOX Transcript Antisense Intergenic RNA
IDC: Invasive ductal carcinoma
JMJD6: Jumanji Domain containing protein 6
J1-C: JMJD6 overexpressing MCF-7 clones
PAM50: Prediction Analysis of Microarray 50
PCDH: Protocadherin
PRC2: Polycomb repressive complex 2
STAT3: Signal transducer and activator of transcription 3
TCGA: The Cancer Genome Atlas
TNBC: Triple negative breast cancer
TSS: Transcription start site
Vec: Empty Vector transfected MCF-7 cells
