# Supplementary material for: Both EZH2 and JMJD6 regulate cell cycle genes in breast cancer
Source: BMC Cancer. 2020 Nov 27;20:1159. doi: 10.1186/s12885-020-07531-8 (PMC7694428; doi:10.1186/s12885-020-07531-8)
Supplement: Supplementary file 1 — Additional file 1. List of primers used for real-time PCR and ChIP-qPCR. [file 12885_2020_7531_MOESM1_ESM.docx]

| **Target** | **Forward (5'-3')** | **Reverse (5'-3')** |
| --- | --- | --- |
|  |  |  |
| **Gene Expression** |  |  |
|  |  |  |
| *ACTB* | GAGCACAGAGCCTCGCCTTT | TCATCATCCATGGTGAGCTGG |
| *JMJD6* | GGTTGACCTTCAGGAGTCCAC | TGCGCTCTTTGCTGACACAGTC |
| *EZH2* | CCCTGACCTCTGTCTTACTTGTGGA | ACGTCAGATGGTGCCAGCAATA |
| *TBP* | GATCAGAACAACAGCCTGCC | TTCTGAATAGGCTGTGGGGT |
|  |  |  |
| **ChIP** |  |  |
|  |  |  |
| ACTB | ACCCACACTGTGCCCATCTACGAG | TCTCCTTAATGTCACGCACGATTTCC |
| SIRT4 | GGCTCAGTGCAGCCTAAATC | AAAATTAGCTGGGCATGGTG |
| AURKA | AGGCTGTGAGATGGCTTGAG | CGATTCTCATGCCTCAGCTT |
| AURKB | CCCGCCTCACGATTAAGGTT | CTAGGTCTTGGCCAGTCAGC |
| ADAM17 | GGCCAGATCCTGTCTCAAAA | CAGCCTCCCAAGTAGCTGAG |
| RAD1&BRIX1 | TAGCCGTACTAGTTTCAGCCAG | GTCTTATTGGAGAACTGACCCCT |
| DNAJC21 | CACCAGGTGTTTGTTCCCTT | TCTTTGTGCCCATCCCACAG |
| IDE | GGTCAGGAGTTGGAGACCAG | CAACCCCTGTCTCTCGTGTT |
| PARP1 | GACAGAGCAATTCCTGTGCAGTG | CCAAGCTACAGGAGCAACTGG |
| NDRG1 | CCACGCTGAAGACCTCAGTT | GGCTCAGCTCACAGGTCTTT |
| CISH | GTCACAGCCAAGAGCTCAGT | CAGGTGAGACAGGCCAGATG |
| IGF2BP3 | GCCTAGGCGACAAGAGTGTT | TGACAAAACTGCTGACAAGCG |
| MYT1 (distal) | AGGCACCTTCTGTTGGCCGA | AGGCAGCTGCCTCCCGTACA |
| MYT1 (proximal) | CAGGAAGACACCTCTCACAC | ACAGTGTCCAGGGGCTTTGC |
