# Supplementary material for: Both EZH2 and JMJD6 regulate cell cycle genes in breast cancer
Source: BMC Cancer. 2020 Nov 27;20:1159. doi: 10.1186/s12885-020-07531-8 (PMC7694428; doi:10.1186/s12885-020-07531-8)
Supplement: Supplementary file 2 — Additional file 2. List of genes co-regulated by both JMJD6 and EZH2 in MCF-7 cells. FC represents Fold Change. [file 12885_2020_7531_MOESM2_ESM.docx]

| **Gene Symbol** | ***JMJD6*** | | ***EZH2*** | |
| --- | --- | --- | --- | --- |
|  | **FC (Log)** | **p-value** | **FC (Log)** | **p-value** |
| *NLGN1* | -0.75142 | 0.00051 | -0.25 | 0.03309 |
| *CITED1* | -3.06827 | 0.00115 | 0.33 | 0.00056 |
| *NGFR* | 3.68133 | 0.00127 | 0.47 | 0.00984 |
| *NXT2* | -2.37493 | 0.00229 | -0.39 | 0.01527 |
| *DDIT4L* | -2.53542 | 0.00289 | 0.18 | 0.01818 |
| *ERCC6L* | -2.0299 | 0.00347 | -0.87 | 0.00509 |
| *FOXM1* | -1.84532 | 0.00407 | -0.48 | 0.00888 |
| *SCG5* | 1.85847 | 0.00408 | 3.38 | 0 |
| *CAV1* | -2.06811 | 0.00482 | -0.64 | 0.00099 |
| *DEPDC1* | -1.87129 | 0.00487 | -1.62 | 0.00017 |
| *SHISA2* | -1.88034 | 0.00563 | 0.6 | 0.00013 |
| *TGFBI* | 1.84025 | 0.00605 | 2.21 | 0.00099 |
| *MGP* | -2.78016 | 0.00629 | 0.17 | 0.01697 |
| *LRP4* | -1.92265 | 0.00641 | 0.17 | 0.00575 |
| *ATP6AP1* | 1.72711 | 0.00676 | 0.78 | 0.03955 |
| *CLDND2* | 1.60883 | 0.00708 | -0.15 | 0.03611 |
| *NCAPD2* | -1.84579 | 0.00766 | -1.12 | 0.00009 |
| *APOLD1* | 1.60614 | 0.00802 | 0.46 | 0.00942 |
| *IFI35* | -1.94723 | 0.0083 | -1.13 | 0.00043 |
| *NDC80* | -1.50582 | 0.00947 | -1.54 | 0.00103 |
| *ADHFE1* | 2.00322 | 0.00953 | -0.12 | 0.01039 |
| *SPC25* | -1.5285 | 0.00984 | -0.96 | 0.01429 |
| *C2CD5* | -1.60752 | 0.01014 | -0.55 | 0.00687 |
| *PIP4K2A* | -1.585 | 0.01026 | -0.46 | 0.00624 |
| *TMEM59L* | 1.56623 | 0.01056 | 0.16 | 0.04405 |
| *HIST1H2BN* | 1.88068 | 0.01135 | -0.13 | 0.0168 |
| *RBM23* | -1.47227 | 0.0128 | -0.59 | 0.00176 |
| *BTBD10* | -1.50545 | 0.01289 | -1.22 | 0.00147 |
| *MELK* | -1.39857 | 0.01315 | -0.93 | 0.00526 |
| *FBXO27* | -1.46366 | 0.0133 | -1.13 | 0.00033 |
| *SLC26A4* | -2.64548 | 0.01378 | -0.15 | 0.00474 |
| *RACGAP1* | -1.36064 | 0.01397 | -0.22 | 0.03913 |
| *PRR3* | 1.12705 | 0.0141 | -0.66 | 0.00008 |
| *TMEM217* | 2.50048 | 0.01429 | 0.31 | 0.00089 |
| *RELN* | -1.55014 | 0.01466 | 0.54 | 0.00676 |
| *SKA1* | -1.70871 | 0.01472 | -0.46 | 0.0093 |
| *RAD51AP1* | -1.33055 | 0.01484 | -0.76 | 0.00161 |
| *CDC45* | -1.36151 | 0.01489 | -0.6 | 0.02295 |
| *ISOC1* | -1.6147 | 0.01502 | -0.97 | 0.00003 |
| *PHTF1* | -2.17849 | 0.01508 | 0.9 | 0.02083 |
| *KBTBD11* | -1.3833 | 0.01517 | 0.28 | 0.03043 |
| *EMG1* | -1.39244 | 0.01543 | -0.99 | 0.00034 |
| *RFC4* | -1.373 | 0.01551 | -1.34 | 0.00012 |
| *C2CD2* | -1.42054 | 0.01551 | 0.35 | 0.00053 |
| *PTTG2* | -1.49171 | 0.01552 | -0.19 | 0.02115 |
| *CCNA2* | -1.36131 | 0.01632 | -1.68 | 0.00059 |
| *NAT9* | 1.3229 | 0.01659 | 0.39 | 0.00466 |
| *CDKN2C* | -1.369 | 0.01679 | -0.34667 | 0.04185 |
| *PIGB* | -1.43357 | 0.0169 | -0.33 | 0.02449 |
| *TBC1D19* | -2.2196 | 0.01708 | 0.39 | 0.0003 |
| *PLA2G4A* | -2.62119 | 0.01728 | -0.89 | 0.0099 |
| *GPX8* | -3.69274 | 0.01748 | -1.17 | 0.00213 |
| *TMEM38A* | 1.77495 | 0.01775 | 0.2 | 0.00312 |
| *SGK1* | -1.38083 | 0.0184 | 2.11 | 0.0006 |
| *LRP8* | -1.37607 | 0.01843 | -0.66 | 0.01618 |
| *ERLIN1* | -1.33162 | 0.01849 | 0.58 | 0.00082 |
| *RAB9B* | -1.23623 | 0.01856 | 0.53 | 0.00342 |
| *MTFR2* | -1.36118 | 0.01857 | -0.72 | 0.00307 |
| *F3* | -1.79167 | 0.01909 | -0.53 | 0.00313 |
| *UHRF1* | -1.28698 | 0.01939 | -0.7 | 0.02762 |
| *GLTSCR2* | 2.22549 | 0.01948 | -0.41 | 0.00108 |
| *HS3ST3A1* | -1.59093 | 0.01979 | 0.33 | 0.01593 |
| *JAZF1* | -1.37088 | 0.02001 | 0.46 | 0.00308 |
| *METTL4* | -1.39123 | 0.02006 | -0.28 | 0.00154 |
| *DFNA5* | 2.23603 | 0.02023 | -0.35 | 0.00598 |
| *ORC1* | -1.22803 | 0.02055 | -0.76 | 0.00054 |
| *BUB1B* | -1.17953 | 0.02093 | -0.68 | 0.02455 |
| *MND1* | -1.21009 | 0.02114 | -1.4 | 0.01595 |
| *RBPMS2* | 1.1685 | 0.02189 | 0.17 | 0.02887 |
| *NEIL3* | -1.23548 | 0.02205 | -0.64 | 0.01624 |
| *NUF2* | -1.16887 | 0.02206 | -0.93 | 0.04423 |
| *AAED1* | -1.29445 | 0.02227 | -0.37 | 0.00603 |
| *SERPINI1* | 1.26037 | 0.02305 | 0.78 | 0.00868 |
| *KCTD20* | -1.30476 | 0.0234 | 0.81 | 0.00519 |
| *CCDC68* | -1.33135 | 0.02384 | 0.2 | 0.00492 |
| *PTDSS2* | -1.77658 | 0.02411 | 0.54 | 0.04622 |
| *KCNS3* | -1.16908 | 0.02469 | 0.56 | 0.00043 |
| *GEMIN2* | -1.14895 | 0.02532 | -0.44 | 0.00033 |
| *HJURP* | -1.1483 | 0.02543 | -1.35 | 0.00029 |
| *PARP2* | -1.14846 | 0.0256 | -0.27 | 0.01821 |
| *MCM7* | -1.15678 | 0.02586 | -0.27667 | 0.02991 |
| *MCRS1* | -1.21732 | 0.02604 | -0.26 | 0.01896 |
| *RFWD3* | -1.13234 | 0.02604 | -0.71 | 0.00066 |
| *MTPN* | 1.12481 | 0.02622 | -0.6 | 0.02236 |
| *HMMR* | -1.1166 | 0.02637 | -0.88 | 0.00593 |
| *KNSTRN* | -1.11691 | 0.02696 | -1.23 | 0.00065 |
| *RAP2C* | -1.33969 | 0.02728 | 0.52 | 0.00059 |
| *SMC2* | -1.22579 | 0.0273 | -0.45 | 0.00732 |
| *CDC20* | -1.08472 | 0.02788 | -2.15 | 0.00006 |
| *CENPM* | -1.11761 | 0.02826 | -1.2 | 0.00042 |
| *POLE2* | -1.14089 | 0.0287 | -0.75 | 0.00017 |
| *HYAL3* | 1.3756 | 0.02963 | 0.45 | 0.01033 |
| *ARMCX2* | -1.13275 | 0.02967 | -0.57 | 0.00003 |
| *ACAT2* | -1.10739 | 0.03043 | -1.25 | 0.0137 |
| *PHLDA1* | -1.63599 | 0.03181 | -0.79 | 0.00415 |
| *CEP55* | -1.03031 | 0.03194 | -1.56 | 0.00108 |
| *FAM64A* | -1.07311 | 0.03259 | -1.15 | 0.00337 |
| *EVA1C* | -1.2115 | 0.03275 | 0.34 | 0.02116 |
| *RMI2* | -1.0281 | 0.03279 | 0.43 | 0.00415 |
| *AURKA* | -1.05256 | 0.03286 | -1.21 | 0.00257 |
| *CHRNA5* | -1.21002 | 0.03378 | -0.32 | 0.04172 |
| *AGPAT5* | -1.33791 | 0.03397 | 0.86 | 0.00001 |
| *COQ3* | -1.17633 | 0.03401 | -0.33 | 0.01416 |
| *MPHOSPH9* | -1.44119 | 0.0345 | -0.49 | 0.00003 |
| *ANLN* | -1.00864 | 0.03469 | -1.07 | 0.00346 |
| *BRIP1* | -1.07495 | 0.03545 | -0.14 | 0.04846 |
| *RPL39L* | -1.23952 | 0.03594 | -1.22 | 0.00224 |
| *NCAPG* | -1.06086 | 0.03706 | -0.8 | 0.00963 |
| *PSMD3* | -1.10584 | 0.03726 | 0.3 | 0.01735 |
| *TMEM17* | -1.25628 | 0.03751 | 0.17 | 0.0137 |
| *ACOX2* | 1.02017 | 0.03775 | -0.77 | 0.00347 |
| *ZBTB4* | -1.17273 | 0.03793 | 0.3 | 0.01194 |
| *PMF1* | -1.01534 | 0.03806 | -0.51 | 0.01992 |
| *CDCA3* | -1.09206 | 0.03872 | -2.12 | 0.00271 |
| *CENPL* | -1.03769 | 0.03877 | -0.57 | 0.02397 |
| *KIF15* | -1.05413 | 0.03897 | -0.79 | 0.03304 |
| *SLCO1B3* | 2.77584 | 0.03922 | -0.57 | 0.00012 |
| *DCTN4* | -1.73475 | 0.03937 | 0.28 | 0.04035 |
| *DCK* | -0.99825 | 0.03944 | -0.98 | 0.02094 |
| *SCNN1D* | -1.00811 | 0.03968 | 0.22 | 0.04487 |
| *RCSD1* | -0.3574 | 0.03984 | -0.46 | 0.00034 |
| *EPDR1* | -1.16822 | 0.04002 | 1.46 | 0.00004 |
| *AOX1* | -0.97659 | 0.04021 | 0.35 | 0.04032 |
| *CTNNAL1* | -0.99069 | 0.04038 | -1.69 | 0.00004 |
| *VPS36* | 0.52128 | 0.04121 | 0.53 | 0.00141 |
| *RABGGTB* | -1.11683 | 0.04125 | -1.61 | 0.00003 |
| *CENPA* | -0.95327 | 0.04137 | -1.21 | 0.00491 |
| *PRADC1* | -1.09037 | 0.04145 | 0.87 | 0.0029 |
| *CDC7* | -0.97684 | 0.04183 | -0.62 | 0.00223 |
| *FUT4* | -1.20142 | 0.04286 | 0.61 | 0.00319 |
| *CYP1A1* | 2.10117 | 0.04309 | 0.23 | 0.03496 |
| *SLC41A1* | 1.08434 | 0.0436 | 0.34 | 0.03946 |
| *C14orf37* | 1.87931 | 0.04381 | 0.86 | 0.00005 |
| *GLI4* | 1.44484 | 0.0439 | 0.15 | 0.00912 |
| *CENPH* | -1.00691 | 0.04403 | -0.5 | 0.01281 |
| *TIPIN* | -1.01004 | 0.04407 | -0.99 | 0.00001 |
| *DHX33* | -0.99899 | 0.0442 | -1.2 | 0.00071 |
| *PAFAH2* | -1.06323 | 0.04435 | -0.21 | 0.02784 |
| *COL6A3* | 1.12055 | 0.0447 | 0.61 | 0.00231 |
| *ZNF17* | 1.21957 | 0.04487 | 0.27 | 0.02999 |
| *GNE* | -1.00138 | 0.04516 | -1.16 | 0.00131 |
| *ARL2* | -1.00359 | 0.04521 | 1.09 | 0.00011 |
| *WDR76* | -1.39173 | 0.04585 | -0.16 | 0.01855 |
| *CAP2* | -0.95991 | 0.04592 | 0.9 | 0.01548 |
| *COL5A1* | 1.35877 | 0.0464 | 0.99 | 0.00001 |
| *PLEK2* | -1.00935 | 0.04644 | 1.32 | 0.00657 |
| *TMEM14A* | -0.91939 | 0.04726 | -0.79 | 0.00109 |
| *ACOT4* | -0.90321 | 0.04827 | -0.19 | 0.00424 |
| *TNFSF13* | -2.88059 | 0.04836 | -0.1 | 0.03342 |
| *IL17D* | -1.3884 | 0.04894 | 0.27 | 0.00207 |
| *PFAS* | -1.11032 | 0.04972 | -0.74 | 0.00208 |
| *MRPL48* | -0.97755 | 0.04978 | -0.73 | 0.00038 |
| *KIF4A* | -0.88814 | 0.04998 | -0.97 | 0.00004 |
| *SLC6A8* | 1.06588 | 0.05017 | 0.15 | 0.04265 |
| *HIST1H3C* | -0.89648 | 0.05091 | -1.25 | 0.00017 |
