# Supplementary material for: Both EZH2 and JMJD6 regulate cell cycle genes in breast cancer
Source: BMC Cancer. 2020 Nov 27;20:1159. doi: 10.1186/s12885-020-07531-8 (PMC7694428; doi:10.1186/s12885-020-07531-8)
Supplement: Supplementary file 3 — Additional file 3. List of genes co-regulated by both JMJD6 and EZH2 in MDA MB 231 cells. FC represents Fold Change. [file 12885_2020_7531_MOESM3_ESM.docx]

| **Gene symbol** | **JMJD6** | | **EZH2** | |
| --- | --- | --- | --- | --- |
|  | **FC (Log)** | **p-value** | **FC (Log)** | **p-value** |
| *Mar-01* | -1.57249 | 0.04125 | -0.25 | 0.00219 |
| *Mar-04* | 0.6194 | 0.05087 | 1.31 | 0.00059 |
| *AAAS* | -1.01567 | 0.02605 | -0.27 | 0.04006 |
| *ABCA3* | 0.6396 | 0.04432 | 1.24 | 0.00005 |
| *ABHD15* | 0.78951 | 0.02504 | 0.26 | 0.04444 |
| *ACOX2* | -1.12406 | 0.01047 | -0.77 | 0.00347 |
| *ACP2* | -0.77292 | 0.03047 | 0.33 | 0.00427 |
| *ADAMTSL5* | -1.30491 | 0.04713 | 0.25 | 0.00922 |
| *ADHFE1* | 0.96001 | 0.02379 | -0.12 | 0.01039 |
| *ADSL* | -0.82592 | 0.03798 | -0.87 | 0.00412 |
| *AGA* | -1.17584 | 0.01151 | 1.02 | 0.00001 |
| *AGPAT5* | -1.40263 | 0.00979 | 0.86 | 0.00001 |
| *AHCY* | -0.92864 | 0.0202 | -0.82 | 0.00008 |
| *AIM2* | 0.83286 | 0.04042 | 0.84 | 0.00031 |
| *AKR1B10* | -2.57494 | 0.00732 | 0.12 | 0.01366 |
| *AKTIP* | 0.79623 | 0.04762 | -0.4 | 0.04302 |
| *ALDH16A1* | -0.9079 | 0.01833 | 0.4 | 0.00134 |
| *ALDH9A1* | 0.80916 | 0.04963 | 0.24 | 0.04645 |
| *AMT* | -1.03174 | 0.0321 | 0.25 | 0.01884 |
| *ANGPTL2* | 1.16305 | 0.02028 | 2.08 | 0 |
| *ANKLE1* | -2.05202 | 0.02305 | 0.1 | 0.02571 |
| *ANKRD13A* | 1.26595 | 0.01107 | -0.59 | 0.00577 |
| *ANKRD29* | 0.86368 | 0.04018 | 0.36 | 0.01611 |
| *ANKRD46* | 1.25399 | 0.01082 | 1.64 | 0.00025 |
| *ANLN* | -0.67157 | 0.04946 | -1.07 | 0.00346 |
| *ANP32B* | -0.76158 | 0.04944 | -0.45 | 0.0008 |
| *AOX1* | -0.98841 | 0.02939 | 0.35 | 0.04032 |
| *APOBEC3B* | -0.78026 | 0.02606 | -0.96 | 0.00006 |
| *APOLD1* | 2.2047 | 0.00205 | 0.46 | 0.00942 |
| *APOO* | -1.06172 | 0.04849 | -0.66 | 0.01146 |
| *AQP11* | -1.3268 | 0.0085 | 0.73 | 0.00171 |
| *ARAF* | -1.13945 | 0.02562 | -0.33 | 0.04475 |
| *ARL1* | -0.7008 | 0.04552 | -0.56 | 0.03899 |
| *ARL4C* | -1.06857 | 0.03782 | 0.42 | 0.03136 |
| *ARL6IP6* | -2.79446 | 0.04098 | -1.93 | 0 |
| *ARNT2* | 0.88482 | 0.0186 | -0.39 | 0.00016 |
| *ARPC1A* | -1.28858 | 0.00776 | 0.55 | 0.00019 |
| *ARRDC3* | 0.94251 | 0.0345 | 0.3 | 0.01636 |
| *ASB13* | -1.40553 | 0.00703 | 0.31 | 0.02073 |
| *ASB9* | -1.24534 | 0.00654 | -0.93 | 0.00011 |
| *ATG2A* | 0.94426 | 0.02706 | 0.4 | 0.01147 |
| *ATP1B1* | 0.97542 | 0.01612 | 0.675 | 0.00312 |
| *ATP6V1E2* | 0.97713 | 0.01526 | -0.24 | 0.02241 |
| *AURKA* | -1.61659 | 0.00613 | -1.21 | 0.00257 |
| *AURKB* | -1.18745 | 0.03381 | -1.74 | 0.00514 |
| *B3GAT3* | 0.72294 | 0.05067 | 0.81 | 0.04509 |
| *BCAT1* | -1.48297 | 0.0158 | -0.66 | 0.00103 |
| *BFAR* | -0.72261 | 0.04331 | -0.61 | 0.00006 |
| *BIK* | 1.68612 | 0.0093 | 0.5 | 0.03477 |
| *BNIP1* | -1.25581 | 0.01363 | -0.35 | 0.02952 |
| *BORA* | -1.47782 | 0.005 | -1.39 | 0.00001 |
| *BRIX1* | -1.09618 | 0.02872 | -0.99 | 0.00003 |
| *BTBD10* | -1.23209 | 0.00844 | -1.22 | 0.00147 |
| *BTN3A2* | -2.24583 | 0.00131 | 0.25 | 0.03395 |
| *BUB1B* | -0.97902 | 0.01686 | -0.68 | 0.02455 |
| *C10orf10* | 1.20413 | 0.03707 | 1.44 | 0.00023 |
| *C10orf35* | -1.1082 | 0.02152 | 0.47 | 0.0002 |
| *C14orf28* | 0.9863 | 0.02483 | 0.74 | 0.00535 |
| *C14orf79* | 2.79806 | 0.00164 | 0.62 | 0.00028 |
| *C15orf48* | -1.96306 | 0.00184 | -2.105 | 0.00006 |
| *C15orf52* | -0.88407 | 0.01906 | 0.47 | 0.00421 |
| *C17orf62* | 1.13058 | 0.01831 | -0.1 | 0.04862 |
| *C1QBP* | -1.38884 | 0.00587 | -0.35 | 0.00911 |
| *C2CD2* | -0.76576 | 0.02898 | 0.35 | 0.00053 |
| *C2CD5* | -1.18181 | 0.00864 | -0.55 | 0.00687 |
| *C2orf47* | -0.76186 | 0.02985 | -1.13 | 0.00035 |
| *CA11* | 1.1736 | 0.00844 | 0.17 | 0.01681 |
| *CABLES2* | 0.94296 | 0.02461 | 0.5 | 0.00051 |
| *CALB2* | -1.27323 | 0.00696 | 3.51 | 0 |
| *CALU* | -0.90748 | 0.03799 | 0.45 | 0.01486 |
| *CAMLG* | 0.66249 | 0.04668 | 0.57 | 0.00355 |
| *CAPNS1* | -1.05723 | 0.01449 | -0.68 | 0.00948 |
| *CARD6* | -1.68714 | 0.00426 | 0.15 | 0.00881 |
| *CAT* | -2.05786 | 0.00867 | 1.01 | 0.04605 |
| *CATSPER1* | -1.17703 | 0.01063 | -0.31 | 0.03002 |
| *CCBE1* | -0.8569 | 0.05064 | 0.21 | 0.00689 |
| *CCDC102A* | -0.74235 | 0.04996 | 0.22 | 0.03632 |
| *CCDC43* | -0.70735 | 0.04983 | -0.35 | 0.00145 |
| *CCDC51* | -0.90755 | 0.03384 | -0.37 | 0.00068 |
| *CCDC92* | -1.01913 | 0.01282 | 1.35 | 0.00205 |
| *CCNA2* | -1.27093 | 0.00928 | -1.68 | 0.00059 |
| *CCNB1* | -1.16645 | 0.0108 | -1.46 | 0.00503 |
| *CCNF* | -0.80917 | 0.05043 | -0.42 | 0.01763 |
| *CCT6B* | 1.28647 | 0.01652 | 0.27 | 0.00493 |
| *CD151* | 0.81828 | 0.02429 | 0.695 | 0.02515 |
| *CD83* | -0.94172 | 0.0371 | -0.44 | 0.00242 |
| *CDC20* | -0.79048 | 0.02696 | -2.15 | 0.00006 |
| *CDC45* | -1.47332 | 0.00899 | -0.6 | 0.02295 |
| *CDC7* | -1.0752 | 0.01674 | -0.62 | 0.00223 |
| *CDCA3* | -1.15541 | 0.01173 | -2.12 | 0.00271 |
| *CDCA4* | -0.66608 | 0.05022 | -0.605 | 0.00583 |
| *CDCA8* | -0.99483 | 0.02168 | -1.24 | 0.00091 |
| *CDKN3* | 0.77413 | 0.04341 | -1.35 | 0.01978 |
| *CDR2L* | 0.93 | 0.02496 | 0.9 | 0.00117 |
| *CENPL* | -0.70813 | 0.04162 | -0.57 | 0.02397 |
| *CENPM* | -1.02519 | 0.01905 | -1.2 | 0.00042 |
| *CEP55* | -0.94104 | 0.01799 | -1.56 | 0.00108 |
| *CHAF1B* | -1.29885 | 0.01059 | -0.8 | 0.00002 |
| *CHST7* | 0.74056 | 0.03934 | 0.46 | 0.00712 |
| *CITED1* | 4.04788 | 0.00911 | 0.33 | 0.00056 |
| *CLEC2B* | -1.24255 | 0.02424 | 0.38 | 0.00996 |
| *CNTNAP1* | 0.9515 | 0.02255 | 0.64 | 0.00124 |
| *COL15A1* | -1.5052 | 0.00434 | -0.18 | 0.00882 |
| *COPS7A* | -1.13734 | 0.00966 | 0.52 | 0.00009 |
| *CPS1* | 0.99585 | 0.02748 | -0.31 | 0.00374 |
| *CRYL1* | 0.86328 | 0.02219 | 0.34 | 0.04353 |
| *CTBS* | -0.9121 | 0.04451 | -0.29 | 0.03428 |
| *CTGF* | -1.34198 | 0.00557 | 1.06 | 0.00175 |
| *CTH* | -0.93515 | 0.02841 | -0.83 | 0.0363 |
| *CTPS1* | -0.9502 | 0.02517 | -0.33 | 0.00156 |
| *CTSH* | 0.9322 | 0.02126 | 0.35 | 0.01904 |
| *CTSO* | 1.05116 | 0.01286 | 0.66 | 0.02074 |
| *CTSV* | 1.4598 | 0.00681 | -0.25 | 0.02503 |
| *CTXN1* | 1.15758 | 0.01519 | 2.52 | 0.00176 |
| *CXCL1* | -1.85494 | 0.00341 | -1.74 | 0.00553 |
| *CXCL16* | 2.03747 | 0.01253 | 0.31 | 0.001 |
| *CXorf38* | -1.22619 | 0.01063 | -0.51 | 0.00091 |
| *CYB561D2* | 1.1609 | 0.05047 | -0.36 | 0.05017 |
| *CYB5R3* | 0.86673 | 0.02084 | -0.62 | 0.01354 |
| *CYP1A1* | 0.80938 | 0.03532 | 0.23 | 0.03496 |
| *CYP1B1* | -2.06908 | 0.00634 | -1.32 | 0.00001 |
| *CYTH1* | -1.31071 | 0.03339 | 0.47 | 0.00018 |
| *DBF4* | -1.05284 | 0.02559 | -0.09 | 0.03105 |
| *DBNDD1* | 2.01596 | 0.02135 | 0.49 | 0.0434 |
| *DCAF12* | -1.46733 | 0.00408 | 0.62 | 0.00883 |
| *DCK* | -1.01167 | 0.01799 | -0.98 | 0.02094 |
| *DCP2* | 0.76416 | 0.04928 | -0.46 | 0.03688 |
| *DCTN4* | -1.27366 | 0.02996 | 0.28 | 0.04035 |
| *DCTN5* | -1.38972 | 0.03366 | 0.61 | 0.02737 |
| *DDIT4* | -1.8821 | 0.00167 | -0.7 | 0.00077 |
| *DDX21* | -1.47166 | 0.00545 | -0.63 | 0.04829 |
| *DEF6* | -0.90284 | 0.02642 | -0.48 | 0.00141 |
| *DEPDC1* | -2.16201 | 0.00234 | -1.62 | 0.00017 |
| *DEXI* | 1.19556 | 0.01629 | 0.55 | 0.0001 |
| *DHX15* | -0.70637 | 0.04975 | -0.74 | 0.00083 |
| *DHX33* | -1.23109 | 0.01045 | -1.2 | 0.00071 |
| *DLD* | -1.42831 | 0.01143 | -0.85 | 0.00571 |
| *DLGAP5* | -0.70645 | 0.04633 | -1.82 | 0.00039 |
| *DNAJB2* | 1.13328 | 0.01229 | 0.75 | 0.00023 |
| *DNM1* | -0.67754 | 0.03807 | 0.12 | 0.01759 |
| *DPYD* | -2.06024 | 0.02428 | -0.92 | 0.00007 |
| *DUSP14* | 0.6953 | 0.04628 | 0.27 | 0.03559 |
| *DUT* | -1.12581 | 0.01388 | -0.13 | 0.00467 |
| *E2F1* | -0.74003 | 0.04856 | -0.32 | 0.00978 |
| *E2F8* | -1.21026 | 0.0202 | -0.58 | 0.00003 |
| *EID2* | 0.92351 | 0.02662 | -0.32 | 0.00663 |
| *EIF2D* | -1.08141 | 0.01347 | -0.86 | 0.00057 |
| *ELOVL7* | 2.06016 | 0.00869 | -0.25 | 0.00493 |
| *EMG1* | -1.2528 | 0.00944 | -0.99 | 0.00034 |
| *ENC1* | 0.91839 | 0.03638 | -0.66 | 0.01563 |
| *ENDOG* | 1.19924 | 0.00896 | 0.57 | 0.01041 |
| *EPDR1* | -0.95361 | 0.033 | 1.46 | 0.00004 |
| *EPHX1* | 0.95074 | 0.04167 | 0.3 | 0.00947 |
| *EPHX4* | -0.7639 | 0.04175 | -0.27 | 0.033 |
| *ERCC6L* | -1.02651 | 0.03743 | -0.87 | 0.00509 |
| *ERLIN1* | -1.98462 | 0.00389 | 0.58 | 0.00082 |
| *ERMP1* | 1.08308 | 0.03167 | 0.2 | 0.0284 |
| *ESPL1* | -1.3957 | 0.02339 | -0.44 | 0.00156 |
| *ETV4* | -1.30484 | 0.00745 | -1.32 | 0.00436 |
| *EVA1C* | -1.09259 | 0.04218 | 0.34 | 0.02116 |
| *EVI2B* | -1.37492 | 0.00524 | -1.19 | 0.00004 |
| *EXOSC7* | -1.06726 | 0.02047 | -0.25 | 0.01616 |
| *EXOSC8* | -0.81795 | 0.02961 | -1.36 | 0.00229 |
| *EZH2* | -0.95937 | 0.03223 | -1.505 | 0.00609 |
| *F2RL1* | -0.66913 | 0.05094 | -1.35 | 0.00001 |
| *FAM213B* | 0.6559 | 0.04973 | 0.81 | 0.02538 |
| *FANCI* | -0.95836 | 0.02689 | -1.03 | 0.00098 |
| *FASTKD3* | -0.82956 | 0.02235 | -0.47 | 0.00282 |
| *FBLN7* | 1.267 | 0.00782 | 0.25 | 0.00269 |
| *FDXR* | 0.69489 | 0.04461 | 0.37 | 0.0036 |
| *FHOD3* | -0.9238 | 0.03412 | -1.29 | 0.00027 |
| *FIBP* | -0.7328 | 0.04063 | -1.15 | 0.00028 |
| *FOLR3* | -0.8603 | 0.0317 | 0.17 | 0.00652 |
| *FOXM1* | -1.56865 | 0.00406 | -0.48 | 0.00888 |
| *FOXO4* | 1.10578 | 0.00977 | 1.22 | 0.01574 |
| *FRK* | 0.94437 | 0.01302 | -0.12 | 0.03733 |
| *G0S2* | -0.65566 | 0.04713 | 0.61 | 0.00001 |
| *GARS* | -0.78796 | 0.02965 | -0.59 | 0.04465 |
| *GEMIN2* | -0.95698 | 0.0214 | -0.44 | 0.00033 |
| *GFPT2* | -0.85608 | 0.02755 | -0.78 | 0.00198 |
| *GJA1* | -1.27604 | 0.01209 | -0.41 | 0.00259 |
| *GJB2* | -1.88196 | 0.00311 | -0.3 | 0.00369 |
| *GLT8D2* | 1.17491 | 0.01214 | 0.37 | 0.00027 |
| *GLYCTK* | -2.59529 | 0.00739 | -0.17 | 0.04718 |
| *GNE* | -0.94077 | 0.01751 | -1.16 | 0.00131 |
| *GPX3* | 0.86443 | 0.02189 | 1.24 | 0.00003 |
| *GPX8* | -0.89901 | 0.02777 | -1.17 | 0.00213 |
| *GRK6* | -1.81872 | 0.00462 | -0.14 | 0.01188 |
| *GSS* | -1.02901 | 0.01797 | -0.25 | 0.04216 |
| *GTPBP2* | 0.15172 | 0.04124 | -0.15 | 0.02037 |
| *HAUS7* | -0.64094 | 0.0494 | -0.715 | 0.0014 |
| *HAUS8* | -0.90265 | 0.04211 | -0.79 | 0.00297 |
| *HECTD3* | -1.23743 | 0.00779 | 0.49 | 0.04686 |
| *HES6* | 2.54147 | 0.00835 | 0.73 | 0.00035 |
| *HIBADH* | -1.12898 | 0.0127 | -0.27 | 0.03914 |
| *HIST1H1C* | 0.7731 | 0.03933 | -2.77 | 0.00044 |
| *HIST1H2AC* | 1.45291 | 0.00409 | -1.61 | 0.00004 |
| *HIST1H2AM* | 2.0429 | 0.00314 | -0.54 | 0.00067 |
| *HIST1H2BC* | 1.05886 | 0.02624 | -0.87 | 0.00691 |
| *HIST1H2BE* | 1.80676 | 0.00247 | -0.77 | 0.04245 |
| *HIST1H2BK* | 1.14853 | 0.01036 | -0.535 | 0.00129 |
| *HIST1H2BO* | 2.07141 | 0.00173 | -0.17 | 0.01083 |
| *HIST1H4A* | 1.45242 | 0.01912 | -0.14 | 0.04942 |
| *HIST2H2BE* | 1.47648 | 0.00368 | 0.43 | 0.03016 |
| *HJURP* | -0.89707 | 0.01731 | -1.35 | 0.00029 |
| *HMGB2* | -1.81732 | 0.01885 | -1.16 | 0.0447 |
| *HN1L* | -0.84765 | 0.04307 | 0.59 | 0.00118 |
| *HRH1* | 0.78017 | 0.04593 | -0.64 | 0.00231 |
| *HSPB1* | 0.68923 | 0.03966 | 1.33 | 0.00392 |
| *IDE* | -1.04932 | 0.03462 | -0.67 | 0.01629 |
| *IFI44* | -1.34151 | 0.0061 | 0.67 | 0.00064 |
| *IFI6* | 1.27268 | 0.01771 | 0.54 | 0.04278 |
| *IGF2BP3* | -1.4795 | 0.02921 | 1.01 | 0.03373 |
| *IGFBP3* | 0.88404 | 0.02679 | 2.78 | 0 |
| *IKBKE* | 0.04379 | 0.03951 | -0.69 | 0.00018 |
| *IL6* | -1.50336 | 0.00721 | -2.64 | 0.00135 |
| *ILF2* | -1.19946 | 0.00885 | -0.45 | 0.03851 |
| *IQCD* | 1.00167 | 0.01387 | -0.11 | 0.01897 |
| *ISOC1* | -2.53122 | 0.00118 | -0.97 | 0.00003 |
| *KAT8* | -1.21897 | 0.02672 | -0.21 | 0.02347 |
| *KCNJ11* | -2.60542 | 0.0213 | 0.15 | 0.03311 |
| *KCNK6* | 0.84179 | 0.05065 | 0.28 | 0.0489 |
| *KCTD20* | -1.49602 | 0.00562 | 0.81 | 0.00519 |
| *KIAA0355* | 0.88301 | 0.03219 | 0.39 | 0.00226 |
| *KIAA0513* | -0.6758 | 0.01981 | 0.56 | 0.00015 |
| *KIAA1024* | 3.85541 | 0.00214 | 0.31 | 0.00059 |
| *KIF14* | -0.71599 | 0.04222 | -1.12 | 0.00316 |
| *KIF15* | -1.32671 | 0.01729 | -0.79 | 0.03304 |
| *KIF20A* | -1.05526 | 0.01508 | -1.4 | 0.00107 |
| *KIF23* | -1.42482 | 0.01083 | -0.715 | 0.01782 |
| *KIF4A* | -0.84541 | 0.0317 | -0.97 | 0.00004 |
| *KIFC1* | -1.30127 | 0.02084 | -0.3 | 0.01621 |
| *KLHL2* | 1.64969 | 0.00564 | -0.98 | 0.00009 |
| *KLHL28* | 0.79348 | 0.04309 | 0.48 | 0.00227 |
| *KNSTRN* | -1.16208 | 0.01255 | -1.23 | 0.00065 |
| *KPNA2* | -0.7686 | 0.04006 | -0.62 | 0.02139 |
| *KRCC1* | 1.92535 | 0.003 | 1.02 | 0.01698 |
| *KRT80* | 1.1801 | 0.01919 | -0.53 | 0.00117 |
| *KRTAP13-1* | -2.05609 | 0.01148 | 0.13 | 0.03775 |
| *LAPTM5* | -1.60458 | 0.00366 | -0.94 | 0.00188 |
| *LCP1* | -0.73652 | 0.03166 | -0.38 | 0.00549 |
| *LGMN* | 1.09072 | 0.01174 | 1.35 | 0.00001 |
| *LMBR1L* | -1.62845 | 0.00539 | 0.74 | 0.00004 |
| *LRP4* | 0.90609 | 0.04885 | 0.17 | 0.00575 |
| *LRP8* | -2.63552 | 0.0034 | -0.66 | 0.01618 |
| *LRRC49* | -0.81697 | 0.0272 | -0.42 | 0.03953 |
| *LSM2* | -0.8198 | 0.04507 | -1.11 | 0.00003 |
| *LZTS3* | 1.49246 | 0.00449 | 0.81 | 0.0055 |
| *MAD2L1* | -0.82977 | 0.04492 | -1.08 | 0.04673 |
| *MAEL* | 0.99945 | 0.01616 | 0.24 | 0.01641 |
| *MAF1* | -0.89038 | 0.02049 | 0.22 | 0.03496 |
| *MAGEA6* | -1.622 | 0.0127 | -0.215 | 0.03477 |
| *MAL2* | -0.98982 | 0.01331 | -0.82 | 0.00009 |
| *MAP1LC3B* | 0.63468 | 0.04993 | 0.89 | 0.00068 |
| *MAPKAPK3* | -1.15947 | 0.01473 | 0.49 | 0.00488 |
| *MCM10* | -0.95829 | 0.03289 | -0.86 | 0 |
| *MCM3* | -0.93703 | 0.01844 | -0.88 | 0.02084 |
| *MCM7* | -1.075 | 0.01529 | -0.27667 | 0.02991 |
| *MCRS1* | -0.87329 | 0.02453 | -0.26 | 0.01896 |
| *MELK* | -1.0243 | 0.0192 | -0.93 | 0.00526 |
| *METAP1* | -0.73206 | 0.03437 | -0.54 | 0.01326 |
| *METTL18* | -0.65287 | 0.04906 | -0.29 | 0.0204 |
| *METTL25* | -0.9158 | 0.01958 | -0.32 | 0.00127 |
| *METTL4* | -1.2135 | 0.01518 | -0.28 | 0.00154 |
| *MFSD1* | -0.62283 | 0.0502 | 0.79 | 0.00037 |
| *MFSD2A* | -0.80892 | 0.04078 | -0.33 | 0.0124 |
| *MFSD6* | 1.36459 | 0.01581 | -0.74 | 0.00045 |
| *MLH1* | -0.69304 | 0.04653 | -0.4 | 0.00595 |
| *MND1* | -0.8528 | 0.03172 | -1.4 | 0.01595 |
| *MOCOS* | -0.85669 | 0.02973 | -0.91 | 0.00024 |
| *MOGS* | 1.62844 | 0.00273 | 0.29 | 0.00813 |
| *MPDU1* | 0.28375 | 0.00954 | -0.45 | 0.01149 |
| *MPHOSPH6* | -0.46553 | 0.02459 | 0.26 | 0.00906 |
| *MPHOSPH9* | -1.58293 | 0.00909 | -0.49 | 0.00003 |
| *MPND* | 0.81473 | 0.025 | -0.31 | 0.00977 |
| *MPP4* | 0.17172 | 0.04204 | -0.16 | 0.02605 |
| *MRPL1* | -0.67155 | 0.05034 | -1.22 | 0.00127 |
| *MRPL3* | -1.19089 | 0.00749 | -0.78 | 0.00015 |
| *MRPL42* | -1.22442 | 0.03653 | -0.8 | 0.0001 |
| *MRPL48* | -1.01019 | 0.0184 | -0.73 | 0.00038 |
| *MRPL9* | -0.67911 | 0.04549 | -0.67 | 0.00005 |
| *MTMR2* | -1.42656 | 0.01392 | 0.27 | 0.0213 |
| *MUTYH* | -0.98411 | 0.01943 | -0.58 | 0.00286 |
| *MXRA7* | 0.99266 | 0.03537 | 0.76 | 0.01762 |
| *MYO5C* | -0.68454 | 0.04768 | -1.02 | 0 |
| *NAF1* | -0.86972 | 0.03474 | -0.27 | 0.01879 |
| *NCAPD2* | -2.30915 | 0.00177 | -1.12 | 0.00009 |
| *NCAPG* | -1.69261 | 0.00544 | -0.8 | 0.00963 |
| *NDC80* | -1.55396 | 0.00455 | -1.54 | 0.00103 |
| *NDRG1* | 1.07844 | 0.0105 | 2.42 | 0.00018 |
| *NDRG3* | -0.81265 | 0.03012 | 1.21 | 0 |
| *NEIL3* | -0.99532 | 0.02542 | -0.64 | 0.01624 |
| *NEXN* | 1.33151 | 0.01217 | 0.46 | 0.03931 |
| *NFE2L1* | 0.81403 | 0.03034 | 0.78 | 0.0054 |
| *NINJ2* | 1.29561 | 0.006 | 0.33 | 0.00013 |
| *NOC3L* | -0.84021 | 0.02727 | -0.75 | 0.01781 |
| *NOX5* | -1.91507 | 0.02686 | -0.38 | 0.00377 |
| *NPAS2* | -1.47989 | 0.00602 | 0.75 | 0.00005 |
| *NPDC1* | -0.69808 | 0.036 | 0.74 | 0.00092 |
| *NQO2* | 0.91446 | 0.02952 | -0.84 | 0.00195 |
| *NR1H3* | -0.81768 | 0.03736 | -0.8 | 0.00005 |
| *NRTN* | 1.50279 | 0.02723 | 0.46 | 0.00142 |
| *NSMCE4A* | -0.96133 | 0.02656 | -0.33 | 0.01686 |
| *NSRP1* | -0.9141 | 0.01772 | -0.4 | 0.00086 |
| *NTN4* | 1.42263 | 0.00421 | 1.1 | 0.00163 |
| *NUBPL* | -1.1301 | 0.0171 | 0.74 | 0.01409 |
| *NUF2* | -1.5502 | 0.00513 | -0.93 | 0.04423 |
| *NUP35* | -1.06725 | 0.01383 | -0.76 | 0.00971 |
| *NXT2* | -1.57987 | 0.00647 | -0.39 | 0.01527 |
| *OARD1* | -0.8687 | 0.03711 | -0.28 | 0.0116 |
| *OMA1* | -1.02442 | 0.0177 | -0.53 | 0.00052 |
| *OPN3* | 2.00154 | 0.00197 | 1.195 | 0.00707 |
| *ORC1* | -1.1203 | 0.01791 | -0.76 | 0.00054 |
| *ORMDL3* | 0.90602 | 0.02745 | 0.61 | 0.02246 |
| *OXSM* | -0.65959 | 0.04477 | -0.26 | 0.00367 |
| *P2RX4* | 1.00581 | 0.01312 | 0.38 | 0.00434 |
| *PAFAH2* | -1.79398 | 0.01238 | -0.21 | 0.02784 |
| *PAG1* | -0.83452 | 0.03653 | 0.41 | 0.0002 |
| *PAN2* | -1.12027 | 0.01497 | -0.18 | 0.02192 |
| *PARP1* | -1.11783 | 0.01414 | -0.78 | 0.00006 |
| *PARP2* | -0.64997 | 0.05078 | -0.27 | 0.01821 |
| *PCCB* | 0.29575 | 0.00499 | -1.14 | 0.00009 |
| *PDCD2L* | -0.84789 | 0.02779 | -1.14 | 0.00007 |
| *PDZD8* | -1.0699 | 0.04262 | -0.37 | 0.02723 |
| *PEX11B* | 0.72033 | 0.04381 | 1.43 | 0.00056 |
| *PFAS* | -1.19981 | 0.00815 | -0.74 | 0.00208 |
| *PGAP2* | -0.77999 | 0.03599 | -0.39 | 0.00105 |
| *PHACTR4* | 0.86054 | 0.03381 | -0.26 | 0.00048 |
| *PHLDA1* | 0.448 | 0.03816 | -0.79 | 0.00415 |
| *PIGB* | -1.40189 | 0.01097 | -0.33 | 0.02449 |
| *PIM1* | -0.90506 | 0.0217 | -0.71 | 0.01749 |
| *PIP4K2A* | -1.88921 | 0.00685 | -0.46 | 0.00624 |
| *PIP5K1C* | 0.80021 | 0.0296 | 1.06 | 0.00048 |
| *PIR* | -2.31938 | 0.00169 | 0.72 | 0.00052 |
| *PIWIL4* | 0.97001 | 0.02517 | 0.11 | 0.03122 |
| *PKMYT1* | -1.04746 | 0.01643 | -0.8 | 0.00002 |
| *PLA2G4C* | 1.6137 | 0.04118 | 0.13 | 0.03506 |
| *PLAC8* | 0.79181 | 0.03074 | -1.44 | 0.01338 |
| *PLCZ1* | -1.89344 | 0.00368 | 0.17 | 0.00373 |
| *PLD5* | 0.35238 | 0.04955 | 1.93 | 0 |
| *PLEKHA4* | 3.84508 | 0.00136 | -0.19 | 0.02177 |
| *PMPCB* | 0.02665 | 0.03503 | -1.05 | 0.00005 |
| *POLA2* | -1.09403 | 0.03487 | -0.4 | 0.00302 |
| *POLD1* | -0.91151 | 0.02855 | -0.6 | 0.00369 |
| *POLE2* | -0.91166 | 0.02513 | -0.75 | 0.00017 |
| *POLR2G* | -0.89094 | 0.01932 | -0.96 | 0.00319 |
| *POT1* | -0.79697 | 0.0358 | -0.36 | 0.0016 |
| *PPFIBP2* | -0.79632 | 0.04039 | -0.4 | 0.00109 |
| *PPP1R35* | -0.72852 | 0.04514 | -1.11 | 0.00178 |
| *PPP1R3C* | -0.13343 | 0.03245 | 0.42 | 0.01468 |
| *PRADC1* | -0.77654 | 0.03598 | 0.87 | 0.0029 |
| *PREB* | -0.67948 | 0.04446 | -0.43 | 0.02757 |
| *PRIM1* | -0.83449 | 0.03 | -1.53 | 0.00231 |
| *PRMT6* | -0.84889 | 0.03149 | -0.35 | 0.01348 |
| *PRR11* | -1.59256 | 0.00443 | -0.75 | 0.04929 |
| *PRR3* | 0.45977 | 0.01948 | -0.66 | 0.00008 |
| *PSG4* | 3.43762 | 0.00162 | 0.18 | 0.00279 |
| *PSMB10* | -1.05959 | 0.02349 | -1.36 | 0.00001 |
| *PSMD3* | -1.116 | 0.02099 | 0.3 | 0.01735 |
| *PSMD9* | 0.8678 | 0.04487 | 0.82 | 0.00031 |
| *PSMG1* | -0.71028 | 0.03991 | -1.23 | 0.00604 |
| *PTGER4* | 1.41 | 0.01036 | -0.68 | 0.00008 |
| *PTGES* | -0.78872 | 0.04992 | -2.2 | 0.00156 |
| *PTTG1* | -0.85903 | 0.02307 | -1.3 | 0.01564 |
| *RAB38* | -1.1983 | 0.00869 | -0.75 | 0.00594 |
| *RAB9B* | -1.03153 | 0.01673 | 0.53 | 0.00342 |
| *RABEPK* | -1.00872 | 0.03825 | -1.25 | 0.00008 |
| *RABGGTB* | -1.11115 | 0.01675 | -1.61 | 0.00003 |
| *RACGAP1* | -1.8831 | 0.00205 | -0.22 | 0.03913 |
| *RAP2C* | -1.82175 | 0.00327 | 0.52 | 0.00059 |
| *RBM23* | -1.17103 | 0.00894 | -0.59 | 0.00176 |
| *RBM28* | -0.66895 | 0.04866 | -0.24 | 0.0005 |
| *REEP6* | 0.8593 | 0.03084 | 0.32 | 0.00653 |
| *RFC4* | -1.46388 | 0.00474 | -1.34 | 0.00012 |
| *RFESD* | -0.94043 | 0.03266 | -0.26 | 0.01864 |
| *RFWD3* | -1.34723 | 0.00492 | -0.71 | 0.00066 |
| *RFX5* | -1.0304 | 0.01322 | -1.11 | 0 |
| *RGS17* | -1.04333 | 0.01264 | 1.38 | 0.00022 |
| *RGS4* | -1.26884 | 0.01331 | 2.23 | 0 |
| *RNF20* | -0.92588 | 0.0209 | -0.59 | 0.00094 |
| *ROR1* | -0.96569 | 0.03301 | 0.39 | 0.01101 |
| *RRAS* | 1.0687 | 0.01075 | 0.96 | 0.00521 |
| *RTN4IP1* | -0.80582 | 0.03702 | -0.71 | 0.00021 |
| *RWDD1* | -1.76062 | 0.02163 | 0.5 | 0.00026 |
| *SCG2* | -1.1571 | 0.03406 | 0.45 | 0.00132 |
| *SCG5* | -0.79739 | 0.03462 | 3.38 | 0 |
| *SDSL* | -1.12554 | 0.01458 | -0.4 | 0.02011 |
| *SERPINI1* | 1.27691 | 0.01055 | 0.78 | 0.00868 |
| *SERTAD1* | 0.84703 | 0.03 | 0.8 | 0.00063 |
| *SESN2* | -0.71482 | 0.03567 | 0.41 | 0.00015 |
| *SGSH* | 1.47628 | 0.01704 | 0.41 | 0.00014 |
| *SHISA2* | -0.72773 | 0.03548 | 0.6 | 0.00013 |
| *SHMT2* | -1.13805 | 0.01056 | -0.45 | 0.02089 |
| *SIPA1L1* | -0.00476 | 0.04428 | -0.99 | 0.03288 |
| *SIRT4* | 3.54056 | 0.01586 | 0.63 | 0.00002 |
| *SKA1* | -1.58371 | 0.00327 | -0.46 | 0.0093 |
| *SKA3* | -1.74925 | 0.02045 | -0.69 | 0.00129 |
| *SLC10A3* | 0.84064 | 0.022 | 0.94 | 0.0001 |
| *SLC12A2* | 1.0826 | 0.01433 | -0.38 | 0.03653 |
| *SLC1A3* | -0.19551 | 0.01498 | -0.55 | 0.01043 |
| *SLC25A43* | -0.76454 | 0.04024 | -0.23 | 0.01307 |
| *SLC30A3* | 1.93287 | 0.02454 | 0.16 | 0.01939 |
| *SLC35B1* | -1.20584 | 0.01816 | 0.26 | 0.04244 |
| *SLC41A1* | 1.194 | 0.01589 | 0.34 | 0.03946 |
| *SLC46A3* | 0.67148 | 0.04394 | 0.79 | 0.0001 |
| *SLC6A8* | 2.83414 | 0.0024 | 0.15 | 0.04265 |
| *SLC7A5* | -2.04825 | 0.00207 | -0.82 | 0.00285 |
| *SLC9A9* | -0.44817 | 0.0239 | 0.11 | 0.04326 |
| *SLFN12* | 1.20772 | 0.01271 | 0.4 | 0.00018 |
| *SMIM7* | -1.08116 | 0.04884 | -1.05 | 0.00001 |
| *SNAPIN* | -0.64591 | 0.04611 | -0.36 | 0.04759 |
| *SNRPA* | -1.41351 | 0.00763 | -0.5 | 0.00186 |
| *SOCS2* | -1.05221 | 0.0496 | -0.68 | 0.0037 |
| *SPATA2* | -0.82044 | 0.02437 | 0.33 | 0.0031 |
| *SPC25* | -1.04634 | 0.01182 | -0.96 | 0.01429 |
| *SPSB1* | 1.0534 | 0.01366 | 0.96 | 0.00069 |
| *SPSB2* | -1.2496 | 0.04474 | -0.26 | 0.01887 |
| *SRP72* | 0.07804 | 0.04547 | -0.72 | 0.00123 |
| *SRXN1* | -0.77764 | 0.03105 | 0.7 | 0.03278 |
| *STEAP1* | -0.69982 | 0.03666 | -0.41 | 0.03285 |
| *STIP1* | -0.71193 | 0.04933 | -0.95 | 0.00031 |
| *SURF4* | 0.90136 | 0.02454 | 0.66 | 0.00027 |
| *SYNCRIP* | -1.61904 | 0.03662 | -1.36 | 0.00032 |
| *SYT1* | -3.20918 | 0.03284 | 0.84 | 0.0008 |
| *TACC3* | -0.72657 | 0.03344 | -1.19 | 0.04207 |
| *TASP1* | -1.88641 | 0.02477 | -0.38 | 0.00059 |
| *TBC1D17* | -0.71547 | 0.03781 | 0.28 | 0.01709 |
| *TBC1D31* | -1.83741 | 0.03138 | -0.8 | 0.00276 |
| *TBC1D7* | 1.70126 | 0.02863 | 0.73 | 0.00499 |
| *TCN1* | -1.29141 | 0.01744 | 1.03 | 0.01032 |
| *TCTA* | -1.28732 | 0.00934 | -0.26 | 0.01508 |
| *TESK2* | -0.93323 | 0.04019 | -0.18 | 0.0471 |
| *TFAP4* | -1.49889 | 0.00413 | -0.14 | 0.00816 |
| *THBD* | -0.86522 | 0.03981 | 0.33 | 0.00023 |
| *THOC6* | -0.98135 | 0.02584 | -0.77 | 0.00064 |
| *TICRR* | -0.98556 | 0.0387 | -0.16 | 0.0302 |
| *TLCD1* | 0.72382 | 0.03191 | -0.68 | 0.00129 |
| *TM7SF2* | 1.17105 | 0.01751 | -0.14 | 0.02135 |
| *TMEM104* | -1.15612 | 0.0323 | 0.33 | 0.0156 |
| *TMEM109* | -0.82651 | 0.02656 | 0.44 | 0.01569 |
| *TMEM126A* | -0.93285 | 0.01742 | -0.56 | 0.04767 |
| *TMEM14A* | -0.79149 | 0.03157 | -0.79 | 0.00109 |
| *TMEM17* | -0.92745 | 0.02416 | 0.17 | 0.0137 |
| *TMEM18* | -0.8979 | 0.04174 | 0.4 | 0.00217 |
| *TMEM184B* | 0.99691 | 0.01494 | 0.56 | 0.00214 |
| *TMEM187* | 1.15232 | 0.00794 | 0.31 | 0.00513 |
| *TMEM2* | 0.78153 | 0.04276 | 1.01 | 0.00111 |
| *TMEM254* | -1.33369 | 0.04516 | 0.65 | 0.01788 |
| *TMEM41A* | 0.72034 | 0.04509 | -0.23 | 0.02535 |
| *TMEM45A* | 0.70231 | 0.03491 | 0.83 | 0.0009 |
| *TMEM62* | -0.10039 | 0.03589 | 0.61 | 0.00266 |
| *TMEM71* | -1.85234 | 0.00275 | -0.98 | 0.00025 |
| *TMEM79* | -0.96307 | 0.04929 | -0.17 | 0.00202 |
| *TMEM86B* | -1.06407 | 0.01002 | 0.46 | 0.0006 |
| *TMEM87A* | -1.0867 | 0.03498 | 0.58 | 0.01094 |
| *TNFAIP8L3* | -0.70514 | 0.04909 | 0.17 | 0.01274 |
| *TNFSF9* | 1.0556 | 0.01282 | 0.33 | 0.03885 |
| *TOP2B* | -0.71341 | 0.0379 | -0.6 | 0.00129 |
| *TOPBP1* | -0.66773 | 0.05021 | -0.44 | 0.04278 |
| *TP53INP2* | 0.70734 | 0.03631 | 0.37 | 0.00199 |
| *TRIB2* | 0.72597 | 0.05086 | -0.4 | 0.04274 |
| *TRIM52* | 1.04549 | 0.01911 | 0.46 | 0.03087 |
| *TRIT1* | -1.17211 | 0.00758 | 0.41 | 0.00524 |
| *TROAP* | -1.09312 | 0.01732 | -0.9 | 0.01197 |
| *TRRAP* | -0.68121 | 0.04061 | -0.47 | 0.0021 |
| *TSEN15* | -0.9009 | 0.02326 | -0.8 | 0.00441 |
| *TSPAN1* | -1.33763 | 0.02861 | -0.17 | 0.03221 |
| *TST* | 0.92884 | 0.01525 | 1.38 | 0.00005 |
| *TTC31* | -0.98516 | 0.0288 | 0.31 | 0.0154 |
| *TTI2* | -0.73031 | 0.03676 | -0.42 | 0.00472 |
| *TTK* | -0.75643 | 0.04374 | -1.35 | 0.00028 |
| *TUBB2A* | 0.7789 | 0.03734 | 1.5 | 0.00002 |
| *TUFT1* | 1.13019 | 0.00919 | 0.92 | 0.00284 |
| *UBE2T* | -1.2512 | 0.00925 | -1.9 | 0.0002 |
| *UFSP2* | -1.19761 | 0.0147 | -0.83 | 0.00003 |
| *UHRF1* | -1.43867 | 0.00835 | -0.7 | 0.02762 |
| *USP16* | -0.98839 | 0.03691 | -0.025 | 0.01763 |
| *UTP18* | -0.92706 | 0.02576 | -1.43 | 0.00011 |
| *VASN* | 2.08169 | 0.00207 | 1.74 | 0 |
| *VPS37B* | 0.72379 | 0.03489 | -1.48 | 0.00792 |
| *VPS54* | -0.7516 | 0.03388 | -0.46 | 0.00288 |
| *VRK2* | -0.84809 | 0.04079 | -0.44 | 0.00245 |
| *WDR6* | 2.01779 | 0.03148 | 0.61 | 0.0041 |
| *WDR76* | -1.09375 | 0.02896 | -0.16 | 0.01855 |
| *WDSUB1* | -0.74784 | 0.05022 | -0.42 | 0.00192 |
| *WT1* | -1.48146 | 0.04331 | -0.34 | 0.01259 |
| *YPEL5* | 1.08248 | 0.01227 | 1.12 | 0.003 |
| *ZBTB4* | -1.1613 | 0.0198 | 0.3 | 0.01194 |
| *ZBTB47* | -0.38202 | 0.04743 | 0.9 | 0.00022 |
| *ZFP3* | 1.10298 | 0.0098 | 0.17 | 0.0194 |
| *ZNF213* | 1.24648 | 0.00628 | 0.17 | 0.00652 |
| *ZNF425* | 2.17906 | 0.02033 | 0.35 | 0.01341 |
| *ZNF432* | 0.88239 | 0.03961 | 0.4 | 0.00119 |
| *ZNF488* | 0.91032 | 0.02285 | -0.59 | 0.00968 |
| *ZNF521* | -0.76255 | 0.02918 | 0.42 | 0.02343 |
| *ZNF558* | 0.85041 | 0.0297 | -0.19 | 0.03402 |
