# Supplementary material for: Both EZH2 and JMJD6 regulate cell cycle genes in breast cancer
Source: BMC Cancer. 2020 Nov 27;20:1159. doi: 10.1186/s12885-020-07531-8 (PMC7694428; doi:10.1186/s12885-020-07531-8)
Supplement: Supplementary file 4 — Additional file 4. Gene Set Enrichment Analysis (GSEA) and the top 20 pathways found in 496 genes. [file 12885_2020_7531_MOESM4_ESM.docx]

| **Gene Set Name** | **# Genes in Gene Set (K)** | **Description** | **# Genes in Overlap** | **k/K** | **p-value** | **FDR** |
| --- | --- | --- | --- | --- | --- | --- |
|  |  |  | **(k)** |  |  | **q-value** |
| FISCHER_DREAM_TARGETS | 929 | Target genes of the DREAM complex. | 113 | 0.12 | 2.00E-89 | 8.00E-86 |
| KINSEY_TARGETS_OF_EWSR1_FLII_FUSION_UP | 1278 | Genes up-regulated in TC71 and EWS502 cells (Ewing's sarcoma) by EWSR1-FLI1 as inferred from RNAi knockdown of this fusion protein. | 109 | 0.09 | 1.00E-69 | 2.00E-66 |
| NUYTTEN_EZH2_TARGETS_DN | 1024 | Genes down-regulated in PC3 cells (prostate cancer) after knockdown of EZH2 by RNAi. | 96 | 0.09 | 8.00E-65 | 1.00E-61 |
| KOBAYASHI_EGFR_SIGNALING_24HR_DN | 251 | Genes down-regulated in H1975 cells NSCLC resistant to gefitinib after treatment with EGFR inhibitor CL-387785 for 24h. | 56 | 0.22 | 5.00E-59 | 5.00E-56 |
| GOBERT_OLIGODENDROCYTE_DIFFERENTIATION_UP | 570 | Genes up-regulated during later stage of differentiation of Oli-Neu cells (oligodendroglial precursor) in response to PD174265 | 71 | 0.12 | 3.00E-56 | 3.00E-53 |
| MARSON_BOUND_BY_E2F4_UNSTIMULATED | 728 | Genes with promoters bound by E2F4 in unstimulated hybridoma cells. | 76 | 0.1 | 2.00E-54 | 2.00E-51 |
| ROSTY_CERVICAL_CANCER_PROLIFERATION_CLUSTER | 140 | The 'Cervical Cancer Proliferation Cluster' (CCPC): genes whose expression in cervical carcinoma positively correlates with that of the HPV E6 and E7 oncogenes; they are also differentially expressed according to disease outcome. | 42 | 0.3 | 2.00E-50 | 1.00E-47 |
| DODD_NASOPHARYNGEAL_CARCINOMA_DN | 1375 | Genes down-regulated in nasopharyngeal carcinoma (NPC) compared to the normal tissue. | 91 | 0.07 | 2.00E-48 | 1.00E-45 |
| SOTIRIOU_BREAST_CANCER_GRADE_1_VS_3_UP | 151 | Up-regulated genes whose expression correlated with histologic grade of invasive breast cancer tumors: comparison of grade 1 vs grade 3. | 41 | 0.27 | 3.00E-47 | 1.00E-44 |
| PUJANA_BRCA1_PCC_NETWORK | 1652 | Genes constituting the BRCA1-PCC network of transcripts whose expression positively correlated (Pearson correlation coefficient, PCC >= 0.4) with that of BRCA1 across a compendium of normal tissues. | 97 | 0.06 | 4.00E-47 | 2.00E-44 |
| SHEDDEN_LUNG_CANCER_POOR_SURVIVAL_A6 | 456 | Cluster 6 of method A: up-regulation of these genes in patients with NSCLC predicts poor survival outcome. | 58 | 0.13 | 2.00E-46 | 9.00E-44 |
| DUTERTRE_ESTRADIOL_RESPONSE_24HR_UP | 324 | Genes up-regulated in MCF7 cells (breast cancer) at 24 h of estradiol treatment. | 50 | 0.15 | 3.00E-44 | 1.00E-41 |
| HORIUCHI_WTAP_TARGETS_DN | 310 | Genes down-regulated in primary endothelial cells (HUVEC) after knockdown of WTAP by RNAi. | 48 | 0.15 | 1.00E-42 | 5.00E-40 |
| BERENJENO_TRANSFORMED_BY_RHOA_UP | 536 | Genes up-regulated in NIH3T3 cells transformed by expression of constitutively active (Q63L) form of RHOA off plasmid vector. | 58 | 0.11 | 2.00E-42 | 8.00E-40 |
| BENPORATH_CYCLING_GENES | 648 | Genes showing cell-cycle stage-specific expression | 62 | 0.1 | 5.00E-42 | 1.00E-39 |
| PUJANA_CHEK2_PCC_NETWORK | 779 | Genes constituting the CHEK2-PCC network of transcripts whose expression positively correlates (Pearson correlation coefficient, PCC >= 0.4) with that of CHEK2 | 66 | 0.08 | 2.00E-41 | 6.00E-39 |
| CHIANG_LIVER_CANCER_SUBCLASS_PROLIFERATION_UP | 178 | Top 200 marker genes up-regulated in the 'proliferation' subclass of hepatocellular carcinoma (HCC); characterized by increased proliferation, high levels of serum AFP and chromosomal instability. | 39 | 0.22 | 5.00E-41 | 2.00E-38 |
| PUJANA_BRCA2_PCC_NETWORK | 423 | Genes constituting the BRCA2-PCC network of transcripts whose expression positively correlated (Pearson correlation coefficient, PCC >= 0.4) with that of BRCA2 across a compendium of normal tissues. | 52 | 0.12 | 7.00E-41 | 2.00E-38 |
| ZHOU_CELL_CYCLE_GENES_IN_IR_RESPONSE_24HR | 128 | Cell cycle genes significantly (p =< 0.05) changed in fibroblast cells at 24 h after exposure to ionizing radiation. | 35 | 0.27 | 1.00E-40 | 3.00E-38 |
| BLUM_RESPONSE_TO_SALIRASIB_DN | 342 | Selected genes down-regulated in response to the Ras inhibitor salirasib in a panel of cancer cell lines with constantly active HRAS | 46 | 0.13 | 5.00E-38 | 1.00E-35 |
