# Supplementary material for: Both EZH2 and JMJD6 regulate cell cycle genes in breast cancer
Source: BMC Cancer. 2020 Nov 27;20:1159. doi: 10.1186/s12885-020-07531-8 (PMC7694428; doi:10.1186/s12885-020-07531-8)
Supplement: Supplementary file 5 — Additional file 5. DREAM target genes co-regulated by both JMJD6 and EZH2 (DOCX 14 kb). [file 12885_2020_7531_MOESM5_ESM.docx]

| **Common genes** | **Exclusive genes** | |
| --- | --- | --- |
|  | **MCF-7** | **MDA-MB-231** |
| *ANLN* | *HMMR* | *AAAS* |
| *AURKA* | *RAD51AP1* | *AGPAT5* |
| *BUB1B* | *FAM64A* | *APOBEC3B* |
| *CCNA2* | *CENPA* | *ARL6IP6* |
| *CDC20* | *SMC2* | *ARRDC3* |
| *CDC45* | *BRIP1* | *AURKB* |
| *CDC7* | *TIPIN* | *BRIX1* |
| *CDCA3* | *CENPH* | *CCNB1* |
| *CENPL* | *PMF1* | *CCNF* |
| *CENPM* | *HIST1H3C* | *CDCA4* |
| *CEP55* | *CDKN2C* | *CDCA8* |
| *DCK* | *RPL39L* | *CDKN3* |
| *DEPDC1* |  | *DBF4* |
| *EMG1* |  | *DCP2* |
| *ERCC6L* |  | *DDX21* |
| *FOXM1* |  | *DHX15* |
| *HJURP* |  | *DLGAP5* |
| *KIF15* |  | *DUT* |
| *KIF4A* |  | *E2F1* |
| *MCM7* |  | *E2F8* |
| *MELK* |  | *ESPL1* |
| *METTL4* |  | *EXOSC8* |
| *MND1* |  | *EZH2* |
| *MPHOSPH9* |  | *FANCI* |
| *NCAPD2* |  | *GARS* |
| *NCAPG* |  | *GRK6* |
| *NDC80* |  | *GTPBP2* |
| *NEIL3* |  | *HAUS7* |
| *NUF2* |  | *HAUS8* |
| *ORC1* |  | *HIST1H1C* |
| *PARP2* |  | *HIST1H2AC* |
| *PFAS* |  | *HIST1H2AM* |
| *POLE2* |  | *HIST1H2BC* |
| *RACGAP1* |  | *HIST1H2BE* |
| *RFC4* |  | *HIST1H2BO* |
| *RFWD3* |  | *HIST1H4A* |
| *SKA1* |  | *HIST2H2BE* |
| *SPC25* |  | *HMGB2* |
| *UHRF1* |  | *KIF14* |
| *WDR76* |  | *KIF20A* |
|  |  | *KIF23* |
|  |  | *KIFC1* |
|  |  | *KPNA2* |
|  |  | *MAD2L1* |
|  |  | *MCM10* |
|  |  | *MCM3* |
|  |  | *MRPL3* |
|  |  | *MUTYH* |
|  |  | *NOC3L* |
|  |  | *NSMCE4A* |
|  |  | *NUP35* |
|  |  | *PGAP2* |
|  |  | *PIM1* |
|  |  | *PKMYT1* |
|  |  | *POLA2* |
|  |  | *POLD1* |
|  |  | *PRIM1* |
|  |  | *PRR11* |
|  |  | *PTTG1* |
|  |  | *SHMT2* |
|  |  | *SKA3* |
|  |  | *SNRPA* |
|  |  | *STIP1* |
|  |  | *SYNCRIP* |
|  |  | *TACC3* |
|  |  | *TFAP4* |
|  |  | *TMEM109* |
|  |  | *TMEM18* |
|  |  | *TOPBP1* |
|  |  | *TROAP* |
|  |  | *TSEN15* |
|  |  | *TTK* |
|  |  | *UBE2T* |
