# Supplementary figures and images for: Both EZH2 and JMJD6 regulate cell cycle genes in breast cancer
Source: BMC Cancer. 2020 Nov 27;20:1159. doi: 10.1186/s12885-020-07531-8 (PMC7694428; doi:10.1186/s12885-020-07531-8)

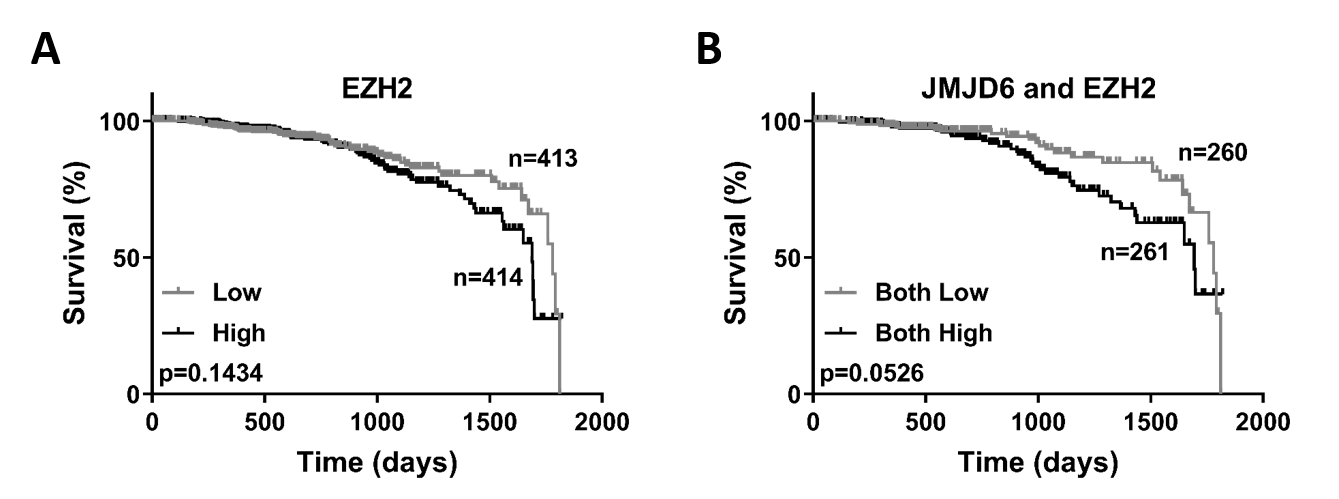

Supplement: Supplementary file 7 — Additional file 7. Survival analysis based on JMJD6 and EZH2 expression. Kaplan Meier curves of overall survival for 5 years for breast cancer patients stratified by median into low and high expressers of EZH2 (A) and combination of JMJD6 and EZH2 (B). [file 12885_2020_7531_MOESM7_ESM.tif]
