# Supplementary material for: Both EZH2 and JMJD6 regulate cell cycle genes in breast cancer
Source: BMC Cancer. 2020 Nov 27;20:1159. doi: 10.1186/s12885-020-07531-8 (PMC7694428; doi:10.1186/s12885-020-07531-8)
Supplement: Supplementary file 8 — Additional file 8. Potential transcription factor binding sites in the conserved JMJD6 binding sites in two clusters types. Cluster with genes IL6, SIRT4, BRIX1 (A), and IGF2BP3, ADAM17, AURKA, Histones, IDE sites (B). The sequence used for PATCH begins with first base of conserved sequence marked in the clusters and PATCH score is given as percent binding. [file 12885_2020_7531_MOESM8_ESM.zip › Additional file 8 AR4.docx]

Identifier Position Mismatches Score Binding Factor Sequence (Search Pattern)

[HS$GG_12](http://gene-regulation.com/cgi-bin/pub/databases/transfac/getTF.cgi?AC=R00558) 14 (-) 0 100.00 [NF-E](http://gene-regulation.com/cgi-bin/pub/databases/transfac/getTF.cgi?AC=T01214) CTGTC

[HS$CYCD1_09](http://gene-regulation.com/cgi-bin/pub/databases/transfac/getTF.cgi?AC=R08644) 35 (+) 0 100.00 [TCF-4E](http://gene-regulation.com/cgi-bin/pub/databases/transfac/getTF.cgi?AC=T02878) TTTTGTT

[HS$PTHRO_03](http://gene-regulation.com/cgi-bin/pub/databases/transfac/getTF.cgi?AC=R12960) 35 (+) 1 90.91 [FOXJ1](http://gene-regulation.com/cgi-bin/pub/databases/transfac/getTF.cgi?AC=T02460) TTTTGTTTGTTT

[HS$ALP_02](http://gene-regulation.com/cgi-bin/pub/databases/transfac/getTF.cgi?AC=R05105) 38 (+) 0 100.00 [HNF-3alpha](http://gene-regulation.com/cgi-bin/pub/databases/transfac/getTF.cgi?AC=T00371), [HNF-3B](http://gene-regulation.com/cgi-bin/pub/databases/transfac/getTF.cgi?AC=T01049) TGTTT

[HS$ADH2_11](http://gene-regulation.com/cgi-bin/pub/databases/transfac/getTF.cgi?AC=R12422) 38 (-) 0 100.00 [GR](http://gene-regulation.com/cgi-bin/pub/databases/transfac/getTF.cgi?AC=T00333) AAAACA

[HS$PTH_04](http://gene-regulation.com/cgi-bin/pub/databases/transfac/getTF.cgi?AC=R11433) 48 (-) 0 100.00 [Ref-1](http://gene-regulation.com/cgi-bin/pub/databases/transfac/getTF.cgi?AC=T04878) GTCTCA

[HS$GG_12](http://gene-regulation.com/cgi-bin/pub/databases/transfac/getTF.cgi?AC=R00558) 65 (+) 0 100.00 [NF-E](http://gene-regulation.com/cgi-bin/pub/databases/transfac/getTF.cgi?AC=T01214) CTGTC

[HS$D1A_01](http://gene-regulation.com/cgi-bin/pub/databases/transfac/getTF.cgi?AC=R09633) 65 (+) 0 100.00 [Meis-2a](http://gene-regulation.com/cgi-bin/pub/databases/transfac/getTF.cgi?AC=T04115), [Meis-2b](http://gene-regulation.com/cgi-bin/pub/databases/transfac/getTF.cgi?AC=T04116), CTGTCA

[Meis-2c](http://gene-regulation.com/cgi-bin/pub/databases/transfac/getTF.cgi?AC=T04117), [Meis-2d](http://gene-regulation.com/cgi-bin/pub/databases/transfac/getTF.cgi?AC=T04118),

[TGIF](http://gene-regulation.com/cgi-bin/pub/databases/transfac/getTF.cgi?AC=T04076)

[HS$UPA_06](http://gene-regulation.com/cgi-bin/pub/databases/transfac/getTF.cgi?AC=R08497) 65 (-) 0 100.00 [Pbx-1a](http://gene-regulation.com/cgi-bin/pub/databases/transfac/getTF.cgi?AC=T01481), [Pbx-1b](http://gene-regulation.com/cgi-bin/pub/databases/transfac/getTF.cgi?AC=T02087), TGACAG

[Pbx-2](http://gene-regulation.com/cgi-bin/pub/databases/transfac/getTF.cgi?AC=T04123), [PKNOX1](http://gene-regulation.com/cgi-bin/pub/databases/transfac/getTF.cgi?AC=T04121),

[PKNOX2](http://gene-regulation.com/cgi-bin/pub/databases/transfac/getTF.cgi?AC=T05155), [UEF-3](http://gene-regulation.com/cgi-bin/pub/databases/transfac/getTF.cgi?AC=T02839)

[HS$LCK_01](http://gene-regulation.com/cgi-bin/pub/databases/transfac/getTF.cgi?AC=R03518) 68 (+) 1 87.50 [LyF-1](http://gene-regulation.com/cgi-bin/pub/databases/transfac/getTF.cgi?AC=T00479) TCTCCCAGG

[HS$IBABP_01](http://gene-regulation.com/cgi-bin/pub/databases/transfac/getTF.cgi?AC=R09992) 68 (-) 0 100.00 [FXR](http://gene-regulation.com/cgi-bin/pub/databases/transfac/getTF.cgi?AC=T04498), [RXR-alpha](http://gene-regulation.com/cgi-bin/pub/databases/transfac/getTF.cgi?AC=T01345) GGGTGA

[HS$OC_03](http://gene-regulation.com/cgi-bin/pub/databases/transfac/getTF.cgi?AC=R10026) 68 (-) 0 100.00 [VDR](http://gene-regulation.com/cgi-bin/pub/databases/transfac/getTF.cgi?AC=T00885) GGGTGA

[HS$BG_01](http://gene-regulation.com/cgi-bin/pub/databases/transfac/getTF.cgi?AC=R00520) 69 (+) 0 100.00 [CACCC-binding factor](http://gene-regulation.com/cgi-bin/pub/databases/transfac/getTF.cgi?AC=T00077) CACCC

[HS$GG_13](http://gene-regulation.com/cgi-bin/pub/databases/transfac/getTF.cgi?AC=R00559) 69 (+) 0 100.00 [gammaCAC1](http://gene-regulation.com/cgi-bin/pub/databases/transfac/getTF.cgi?AC=T00074), CACCC

[gammaCAC2](http://gene-regulation.com/cgi-bin/pub/databases/transfac/getTF.cgi?AC=T00075)

[HS$GG_14](http://gene-regulation.com/cgi-bin/pub/databases/transfac/getTF.cgi?AC=R00560) 69 (+) 0 100.00 [CACCC-binding factor](http://gene-regulation.com/cgi-bin/pub/databases/transfac/getTF.cgi?AC=T00077) CACCC

[Sp1](http://gene-regulation.com/cgi-bin/pub/databases/transfac/getTF.cgi?AC=T00759)

[HS$GP2B_01](http://gene-regulation.com/cgi-bin/pub/databases/transfac/getTF.cgi?AC=R00596) 69 (+) 0 100.00 [CACCC-binding factor](http://gene-regulation.com/cgi-bin/pub/databases/transfac/getTF.cgi?AC=T00077) CACCC

[HS$EG_06](http://gene-regulation.com/cgi-bin/pub/databases/transfac/getTF.cgi?AC=R03118) 69 (+) 0 100.00 [Sp1](http://gene-regulation.com/cgi-bin/pub/databases/transfac/getTF.cgi?AC=T00759) CACCC

[HS$MT2A_11](http://gene-regulation.com/cgi-bin/pub/databases/transfac/getTF.cgi?AC=R08293) 80 (-) 0 100.00 [MTF-1](http://gene-regulation.com/cgi-bin/pub/databases/transfac/getTF.cgi?AC=T02354) TGCACTC

[HS$CRP_03](http://gene-regulation.com/cgi-bin/pub/databases/transfac/getTF.cgi?AC=R00239) 86 (+) 0 100.00 [C/EBPalpha](http://gene-regulation.com/cgi-bin/pub/databases/transfac/getTF.cgi?AC=T00105), AGTGGCGCAA

[C/EBPbeta](http://gene-regulation.com/cgi-bin/pub/databases/transfac/getTF.cgi?AC=T00581),

[C/EBPdelta](http://gene-regulation.com/cgi-bin/pub/databases/transfac/getTF.cgi?AC=T00109)

[HS$FN_01](http://gene-regulation.com/cgi-bin/pub/databases/transfac/getTF.cgi?AC=R00454) 87 (+) 1 88.89 [CREB](http://gene-regulation.com/cgi-bin/pub/databases/transfac/getTF.cgi?AC=T00163) GTGACGCAAT

[HS$GG_26](http://gene-regulation.com/cgi-bin/pub/databases/transfac/getTF.cgi?AC=R02826) 93 (-) 0 100.00 [GATA-1](http://gene-regulation.com/cgi-bin/pub/databases/transfac/getTF.cgi?AC=T00306) AGATTG

[HS$INSR_01](http://gene-regulation.com/cgi-bin/pub/databases/transfac/getTF.cgi?AC=R03068) 102 (-) 1 87.50 [C/EBPalpha](http://gene-regulation.com/cgi-bin/pub/databases/transfac/getTF.cgi?AC=T00108) TGCAGTAAG

[HS$TERT_03](http://gene-regulation.com/cgi-bin/pub/databases/transfac/getTF.cgi?AC=R09485) 113 (+) 1 87.50 [Sp1](http://gene-regulation.com/cgi-bin/pub/databases/transfac/getTF.cgi?AC=T00759) CTCCGCCTC

[HS$GX_WT1_02](http://gene-regulation.com/cgi-bin/pub/databases/transfac/getTF.cgi?AC=R02308) 115 (+) 1 87.50 [WT1 -KTS](http://gene-regulation.com/cgi-bin/pub/databases/transfac/getTF.cgi?AC=T01839), [WT1 I](http://gene-regulation.com/cgi-bin/pub/databases/transfac/getTF.cgi?AC=T01840), CTCCCTCCC

[WT1 I -KTS](http://gene-regulation.com/cgi-bin/pub/databases/transfac/getTF.cgi?AC=T00900),

[WT1 I-del2](http://gene-regulation.com/cgi-bin/pub/databases/transfac/getTF.cgi?AC=T01842),

[WT1-del2](http://gene-regulation.com/cgi-bin/pub/databases/transfac/getTF.cgi?AC=T01841)

[HS$CD8A_02](http://gene-regulation.com/cgi-bin/pub/databases/transfac/getTF.cgi?AC=R08266) 118 (+) 1 87.50 [LyF-1](http://gene-regulation.com/cgi-bin/pub/databases/transfac/getTF.cgi?AC=T02702) CCTCCCAAG

[HS$APOB_10](http://gene-regulation.com/cgi-bin/pub/databases/transfac/getTF.cgi?AC=R03661) 119 (-) 0 100.00 [AP-2alphaA](http://gene-regulation.com/cgi-bin/pub/databases/transfac/getTF.cgi?AC=T00035), TGGGAG

[AP-2alphaB](http://gene-regulation.com/cgi-bin/pub/databases/transfac/getTF.cgi?AC=T02466)

[HS$TLN_01](http://gene-regulation.com/cgi-bin/pub/databases/transfac/getTF.cgi?AC=R11007) 120 (+) 0 100.00 [LUN-1](http://gene-regulation.com/cgi-bin/pub/databases/transfac/getTF.cgi?AC=T04734) TCCCA

[STAT5A](http://gene-regulation.com/cgi-bin/pub/databases/transfac/getTF.cgi?AC=T04683), [STAT5B](http://gene-regulation.com/cgi-bin/pub/databases/transfac/getTF.cgi?AC=T04761),

[STAT6](http://gene-regulation.com/cgi-bin/pub/databases/transfac/getTF.cgi?AC=T01580)

[HS$CDH1_01](http://gene-regulation.com/cgi-bin/pub/databases/transfac/getTF.cgi?AC=R11006) 120 (-) 0 100.00 [LUN-1](http://gene-regulation.com/cgi-bin/pub/databases/transfac/getTF.cgi?AC=T04734) TGGGA

[HS$P21WAF1_05](http://gene-regulation.com/cgi-bin/pub/databases/transfac/getTF.cgi?AC=R10031) 125 (+) 0 100.00 [RXR-alpha](http://gene-regulation.com/cgi-bin/pub/databases/transfac/getTF.cgi?AC=T01345), [VDR](http://gene-regulation.com/cgi-bin/pub/databases/transfac/getTF.cgi?AC=T00885) GGTTCA

[HS$ADH3_01](http://gene-regulation.com/cgi-bin/pub/databases/transfac/getTF.cgi?AC=R03017) 126 (-) 0 100.00 [CAR](http://gene-regulation.com/cgi-bin/pub/databases/transfac/getTF.cgi?AC=T02261), [RAR-alpha1](http://gene-regulation.com/cgi-bin/pub/databases/transfac/getTF.cgi?AC=T00719), TGAAC

[RAR-beta](http://gene-regulation.com/cgi-bin/pub/databases/transfac/getTF.cgi?AC=T00721),

[RXR-alpha](http://gene-regulation.com/cgi-bin/pub/databases/transfac/getTF.cgi?AC=T01345)

[HS$PLOD1_02](http://gene-regulation.com/cgi-bin/pub/databases/transfac/getTF.cgi?AC=R11013) 161 (+) 2 88.89 [PITX2](http://gene-regulation.com/cgi-bin/pub/databases/transfac/getTF.cgi?AC=T02413) GCTGGGATTACAGGTGTGAG

[HS$TLN_01](http://gene-regulation.com/cgi-bin/pub/databases/transfac/getTF.cgi?AC=R11007) 163 (-) 0 100.00 [LUN-1](http://gene-regulation.com/cgi-bin/pub/databases/transfac/getTF.cgi?AC=T04734) TCCCA

[HS$ALBU_03](http://gene-regulation.com/cgi-bin/pub/databases/transfac/getTF.cgi?AC=R00079) 179 (-) 0 100.00 [NF-1/L](http://gene-regulation.com/cgi-bin/pub/databases/transfac/getTF.cgi?AC=T00599) TGGCA

[HS$BG_48](http://gene-regulation.com/cgi-bin/pub/databases/transfac/getTF.cgi?AC=R04295) 181 (-) 0 100.00 [CAC-binding protein](http://gene-regulation.com/cgi-bin/pub/databases/transfac/getTF.cgi?AC=T00076) GGTGG

[HS$CXCR4_02](http://gene-regulation.com/cgi-bin/pub/databases/transfac/getTF.cgi?AC=R09477) 189 (+) 0 100.00 [c-Myc](http://gene-regulation.com/cgi-bin/pub/databases/transfac/getTF.cgi?AC=T00140), [USF2](http://gene-regulation.com/cgi-bin/pub/databases/transfac/getTF.cgi?AC=T00878) CACCTG

[HS$GRH_01](http://gene-regulation.com/cgi-bin/pub/databases/transfac/getTF.cgi?AC=R00607) 237 (+) 0 100.00 [AP-2](http://gene-regulation.com/cgi-bin/pub/databases/transfac/getTF.cgi?AC=T00034), [AP-2alphaA](http://gene-regulation.com/cgi-bin/pub/databases/transfac/getTF.cgi?AC=T00035), TGGCC

[AP-2alphaB](http://gene-regulation.com/cgi-bin/pub/databases/transfac/getTF.cgi?AC=T02466), [NF-1](http://gene-regulation.com/cgi-bin/pub/databases/transfac/getTF.cgi?AC=T00535)

[HS$GRH_01](http://gene-regulation.com/cgi-bin/pub/databases/transfac/getTF.cgi?AC=R00607) 238 (+) 0 100.00 [AP-2](http://gene-regulation.com/cgi-bin/pub/databases/transfac/getTF.cgi?AC=T00034), [AP-2alphaA](http://gene-regulation.com/cgi-bin/pub/databases/transfac/getTF.cgi?AC=T00035), GGCCA

[AP-2alphaB](http://gene-regulation.com/cgi-bin/pub/databases/transfac/getTF.cgi?AC=T02466), [NF-1](http://gene-regulation.com/cgi-bin/pub/databases/transfac/getTF.cgi?AC=T00535)

[HS$TGFB1_04](http://gene-regulation.com/cgi-bin/pub/databases/transfac/getTF.cgi?AC=R01729) 245 (-) 1 87.50 [AP-1](http://gene-regulation.com/cgi-bin/pub/databases/transfac/getTF.cgi?AC=T00029), [Sp1](http://gene-regulation.com/cgi-bin/pub/databases/transfac/getTF.cgi?AC=T00759) TGAGACGAG

[HS$PTH_04](http://gene-regulation.com/cgi-bin/pub/databases/transfac/getTF.cgi?AC=R11433) 248 (-) 0 100.00 [Ref-1](http://gene-regulation.com/cgi-bin/pub/databases/transfac/getTF.cgi?AC=T04878) TGAGAC

[RAR-beta](http://gene-regulation.com/cgi-bin/pub/databases/transfac/getTF.cgi?AC=T00721),

[RXR-alpha](http://gene-regulation.com/cgi-bin/pub/databases/transfac/getTF.cgi?AC=T01345)

[HS$SHP1_01](http://gene-regulation.com/cgi-bin/pub/databases/transfac/getTF.cgi?AC=R10077) 260 (+) 0 100.00 [FXR](http://gene-regulation.com/cgi-bin/pub/databases/transfac/getTF.cgi?AC=T04498), [RXR-alpha](http://gene-regulation.com/cgi-bin/pub/databases/transfac/getTF.cgi?AC=T01345) TGACCT

[HS$CYP3A4_01](http://gene-regulation.com/cgi-bin/pub/databases/transfac/getTF.cgi?AC=R10090) 260 (-) 0 100.00 [PXR-1](http://gene-regulation.com/cgi-bin/pub/databases/transfac/getTF.cgi?AC=T04617), [RXR-alpha](http://gene-regulation.com/cgi-bin/pub/databases/transfac/getTF.cgi?AC=T01345), AGGTCA

[RXR-beta](http://gene-regulation.com/cgi-bin/pub/databases/transfac/getTF.cgi?AC=T01334), [SXR](http://gene-regulation.com/cgi-bin/pub/databases/transfac/getTF.cgi?AC=T04610)

[PPAR-gamma](http://gene-regulation.com/cgi-bin/pub/databases/transfac/getTF.cgi?AC=T02529),

[RXR-alpha](http://gene-regulation.com/cgi-bin/pub/databases/transfac/getTF.cgi?AC=T01345)

[CAC-binding protein](http://gene-regulation.com/cgi-bin/pub/databases/transfac/getTF.cgi?AC=T00076)

[CAC-binding protein](http://gene-regulation.com/cgi-bin/pub/databases/transfac/getTF.cgi?AC=T00076)

[HS$CXCR4_02](http://gene-regulation.com/cgi-bin/pub/databases/transfac/getTF.cgi?AC=R09477) 275 (+) 0 100.00 [c-Myc](http://gene-regulation.com/cgi-bin/pub/databases/transfac/getTF.cgi?AC=T00140), [USF2](http://gene-regulation.com/cgi-bin/pub/databases/transfac/getTF.cgi?AC=T00878) CACCTG

[HS$GG_20](http://gene-regulation.com/cgi-bin/pub/databases/transfac/getTF.cgi?AC=R01859) 280 (+) 0 100.00 [CP1](http://gene-regulation.com/cgi-bin/pub/databases/transfac/getTF.cgi?AC=T00150), [NF-E3](http://gene-regulation.com/cgi-bin/pub/databases/transfac/getTF.cgi?AC=T00559) GCCTTG

[HS$ADH3_01](http://gene-regulation.com/cgi-bin/pub/databases/transfac/getTF.cgi?AC=R03017) 284 (+) 0 100.00 [CAR](http://gene-regulation.com/cgi-bin/pub/databases/transfac/getTF.cgi?AC=T02261), [RAR-alpha1](http://gene-regulation.com/cgi-bin/pub/databases/transfac/getTF.cgi?AC=T00719), TGACC

[RAR-beta](http://gene-regulation.com/cgi-bin/pub/databases/transfac/getTF.cgi?AC=T00721),

[RXR-alpha](http://gene-regulation.com/cgi-bin/pub/databases/transfac/getTF.cgi?AC=T01345)

[HS$SHP1_01](http://gene-regulation.com/cgi-bin/pub/databases/transfac/getTF.cgi?AC=R10077) 284 (+) 0 100.00 [FXR](http://gene-regulation.com/cgi-bin/pub/databases/transfac/getTF.cgi?AC=T04498), [RXR-alpha](http://gene-regulation.com/cgi-bin/pub/databases/transfac/getTF.cgi?AC=T01345) TGACCT

[HS$CYP3A4_01](http://gene-regulation.com/cgi-bin/pub/databases/transfac/getTF.cgi?AC=R10090) 284 (-) 0 100.00 [PXR-1](http://gene-regulation.com/cgi-bin/pub/databases/transfac/getTF.cgi?AC=T04617), [RXR-alpha](http://gene-regulation.com/cgi-bin/pub/databases/transfac/getTF.cgi?AC=T01345), AGGTCA

[RXR-beta](http://gene-regulation.com/cgi-bin/pub/databases/transfac/getTF.cgi?AC=T01334), [SXR](http://gene-regulation.com/cgi-bin/pub/databases/transfac/getTF.cgi?AC=T04610)

[HS$LCK_02](http://gene-regulation.com/cgi-bin/pub/databases/transfac/getTF.cgi?AC=R03519) 287 (+) 1 87.50 [LyF-1](http://gene-regulation.com/cgi-bin/pub/databases/transfac/getTF.cgi?AC=T00479) CCTCCCAAC

[HS$APOB_10](http://gene-regulation.com/cgi-bin/pub/databases/transfac/getTF.cgi?AC=R03661) 288 (-) 0 100.00 [AP-2alphaA](http://gene-regulation.com/cgi-bin/pub/databases/transfac/getTF.cgi?AC=T00035), TGGGAG

[AP-2alphaB](http://gene-regulation.com/cgi-bin/pub/databases/transfac/getTF.cgi?AC=T02466)

[HS$CDH1_01](http://gene-regulation.com/cgi-bin/pub/databases/transfac/getTF.cgi?AC=R11006) 289 (+) 0 100.00 [LUN-1](http://gene-regulation.com/cgi-bin/pub/databases/transfac/getTF.cgi?AC=T04734) TCCCA

[HS$HIOMTA_01](http://gene-regulation.com/cgi-bin/pub/databases/transfac/getTF.cgi?AC=R09056) 302 (+) 0 100.00 [Crx](http://gene-regulation.com/cgi-bin/pub/databases/transfac/getTF.cgi?AC=T03458) GGATTAC

[HS$GHA_05](http://gene-regulation.com/cgi-bin/pub/databases/transfac/getTF.cgi?AC=R00593) 304 (+) 0 100.00 [GR](http://gene-regulation.com/cgi-bin/pub/databases/transfac/getTF.cgi?AC=T00333), [GR-alpha](http://gene-regulation.com/cgi-bin/pub/databases/transfac/getTF.cgi?AC=T00337), ATTACA

[GR-beta](http://gene-regulation.com/cgi-bin/pub/databases/transfac/getTF.cgi?AC=T01920)

[HS$ADH2_10](http://gene-regulation.com/cgi-bin/pub/databases/transfac/getTF.cgi?AC=R12421) 304 (+) 0 100.00 [GR](http://gene-regulation.com/cgi-bin/pub/databases/transfac/getTF.cgi?AC=T00333) ATTACA

[HS$GG_33](http://gene-regulation.com/cgi-bin/pub/databases/transfac/getTF.cgi?AC=R02888) 329 (+) 0 100.00 [CP1](http://gene-regulation.com/cgi-bin/pub/databases/transfac/getTF.cgi?AC=T00150), [NFE-6](http://gene-regulation.com/cgi-bin/pub/databases/transfac/getTF.cgi?AC=T01216) AACCAAT

[HS$BAC_03](http://gene-regulation.com/cgi-bin/pub/databases/transfac/getTF.cgi?AC=R00039) 331 (+) 0 100.00 [SRF](http://gene-regulation.com/cgi-bin/pub/databases/transfac/getTF.cgi?AC=T00764) CCAAT

[HS$GG_15](http://gene-regulation.com/cgi-bin/pub/databases/transfac/getTF.cgi?AC=R00561) 331 (+) 0 100.00 [NF-E](http://gene-regulation.com/cgi-bin/pub/databases/transfac/getTF.cgi?AC=T00556) CCAAT

[HS$GG_16](http://gene-regulation.com/cgi-bin/pub/databases/transfac/getTF.cgi?AC=R00562) 331 (+) 0 100.00 [CDP2](http://gene-regulation.com/cgi-bin/pub/databases/transfac/getTF.cgi?AC=T02043), [Clox](http://gene-regulation.com/cgi-bin/pub/databases/transfac/getTF.cgi?AC=T01995), [CUTL1](http://gene-regulation.com/cgi-bin/pub/databases/transfac/getTF.cgi?AC=T00100), CCAAT

[Cutl1](http://gene-regulation.com/cgi-bin/pub/databases/transfac/getTF.cgi?AC=T02042)

[HS$GG_17](http://gene-regulation.com/cgi-bin/pub/databases/transfac/getTF.cgi?AC=R00563) 331 (+) 0 100.00 [gammaCAAT](http://gene-regulation.com/cgi-bin/pub/databases/transfac/getTF.cgi?AC=T00073) CCAAT

[HS$GG_18](http://gene-regulation.com/cgi-bin/pub/databases/transfac/getTF.cgi?AC=R00564) 331 (+) 0 100.00 [CP1](http://gene-regulation.com/cgi-bin/pub/databases/transfac/getTF.cgi?AC=T00150) CCAAT

[HS$ZG_07](http://gene-regulation.com/cgi-bin/pub/databases/transfac/getTF.cgi?AC=R00571) 331 (+) 0 100.00 [CP2](http://gene-regulation.com/cgi-bin/pub/databases/transfac/getTF.cgi?AC=T00151) CCAAT

[HS$HH1_03](http://gene-regulation.com/cgi-bin/pub/databases/transfac/getTF.cgi?AC=R00660) 331 (+) 0 100.00 [H1TF2](http://gene-regulation.com/cgi-bin/pub/databases/transfac/getTF.cgi?AC=T00388) CCAAT

[HS$GG_20](http://gene-regulation.com/cgi-bin/pub/databases/transfac/getTF.cgi?AC=R01859) 331 (+) 0 100.00 [CP1](http://gene-regulation.com/cgi-bin/pub/databases/transfac/getTF.cgi?AC=T00150), [NF-E3](http://gene-regulation.com/cgi-bin/pub/databases/transfac/getTF.cgi?AC=T00559) CCAAT

[HS$GG_21](http://gene-regulation.com/cgi-bin/pub/databases/transfac/getTF.cgi?AC=R02048) 331 (+) 0 100.00 [CP1](http://gene-regulation.com/cgi-bin/pub/databases/transfac/getTF.cgi?AC=T01202) CCAAT

[HS$GHA_10](http://gene-regulation.com/cgi-bin/pub/databases/transfac/getTF.cgi?AC=R02848) 331 (+) 0 100.00 [alpha-CBF](http://gene-regulation.com/cgi-bin/pub/databases/transfac/getTF.cgi?AC=T00081) CCAAT

[HS$EG_07](http://gene-regulation.com/cgi-bin/pub/databases/transfac/getTF.cgi?AC=R03119) 331 (+) 0 100.00 [CP1](http://gene-regulation.com/cgi-bin/pub/databases/transfac/getTF.cgi?AC=T00150) CCAAT

[HS$CYBH_01](http://gene-regulation.com/cgi-bin/pub/databases/transfac/getTF.cgi?AC=R03477) 331 (+) 0 100.00 [CDP2](http://gene-regulation.com/cgi-bin/pub/databases/transfac/getTF.cgi?AC=T02043), [Clox](http://gene-regulation.com/cgi-bin/pub/databases/transfac/getTF.cgi?AC=T01995), [CP1](http://gene-regulation.com/cgi-bin/pub/databases/transfac/getTF.cgi?AC=T00150), [CUTL1](http://gene-regulation.com/cgi-bin/pub/databases/transfac/getTF.cgi?AC=T00100), CCAAT

[Cutl1](http://gene-regulation.com/cgi-bin/pub/databases/transfac/getTF.cgi?AC=T02042)

[HS$GHA_06](http://gene-regulation.com/cgi-bin/pub/databases/transfac/getTF.cgi?AC=R00594) 331 (-) 0 100.00 [NF-1](http://gene-regulation.com/cgi-bin/pub/databases/transfac/getTF.cgi?AC=T00534) ATTGG

[HS$HSP70_02](http://gene-regulation.com/cgi-bin/pub/databases/transfac/getTF.cgi?AC=R00761) 331 (-) 0 100.00 [CTF](http://gene-regulation.com/cgi-bin/pub/databases/transfac/getTF.cgi?AC=T00174) ATTGG

[HS$HSP70_07](http://gene-regulation.com/cgi-bin/pub/databases/transfac/getTF.cgi?AC=R00766) 331 (-) 0 100.00 [CBTF](http://gene-regulation.com/cgi-bin/pub/databases/transfac/getTF.cgi?AC=T01373), [CP1](http://gene-regulation.com/cgi-bin/pub/databases/transfac/getTF.cgi?AC=T00150), [CTF](http://gene-regulation.com/cgi-bin/pub/databases/transfac/getTF.cgi?AC=T00174) ATTGG

[HS$TK_02](http://gene-regulation.com/cgi-bin/pub/databases/transfac/getTF.cgi?AC=R03040) 331 (-) 0 100.00 [CP1](http://gene-regulation.com/cgi-bin/pub/databases/transfac/getTF.cgi?AC=T01171) ATTGG

[HS$CDC2_07](http://gene-regulation.com/cgi-bin/pub/databases/transfac/getTF.cgi?AC=R04344) 331 (-) 0 100.00 [CBAF](http://gene-regulation.com/cgi-bin/pub/databases/transfac/getTF.cgi?AC=T05107), [CBF-B](http://gene-regulation.com/cgi-bin/pub/databases/transfac/getTF.cgi?AC=T00088), [CP1A](http://gene-regulation.com/cgi-bin/pub/databases/transfac/getTF.cgi?AC=T00154) ATTGG

[HS$CDC2_12](http://gene-regulation.com/cgi-bin/pub/databases/transfac/getTF.cgi?AC=R12441) 331 (-) 0 100.00 [CBAF](http://gene-regulation.com/cgi-bin/pub/databases/transfac/getTF.cgi?AC=T05107) ATTGG
