# Supplementary material for: Both EZH2 and JMJD6 regulate cell cycle genes in breast cancer
Source: BMC Cancer. 2020 Nov 27;20:1159. doi: 10.1186/s12885-020-07531-8 (PMC7694428; doi:10.1186/s12885-020-07531-8)
Supplement: Supplementary file 8 — Additional file 8. Potential transcription factor binding sites in the conserved JMJD6 binding sites in two clusters types. Cluster with genes IL6, SIRT4, BRIX1 (A), and IGF2BP3, ADAM17, AURKA, Histones, IDE sites (B). The sequence used for PATCH begins with first base of conserved sequence marked in the clusters and PATCH score is given as percent binding. [file 12885_2020_7531_MOESM8_ESM.zip › Additional file 8 BR4.docx]

Identifier Position Mismatches Score Binding Factor Sequence (Search Pattern)

[HS$ADH2_11](http://gene-regulation.com/cgi-bin/pub/databases/transfac/getTF.cgi?AC=R12422) 2 (+) 0 100.00 [GR](http://gene-regulation.com/cgi-bin/pub/databases/transfac/getTF.cgi?AC=T00333) AAAACA

[HS$HH1_01](http://gene-regulation.com/cgi-bin/pub/databases/transfac/getTF.cgi?AC=R00658) 3 (+) 0 100.00 [HiNF-A](http://gene-regulation.com/cgi-bin/pub/databases/transfac/getTF.cgi?AC=T00360) AAACACA

[HS$ALP_02](http://gene-regulation.com/cgi-bin/pub/databases/transfac/getTF.cgi?AC=R05105) 3 (-) 0 100.00 [HNF-3alpha](http://gene-regulation.com/cgi-bin/pub/databases/transfac/getTF.cgi?AC=T00371), [HNF-3B](http://gene-regulation.com/cgi-bin/pub/databases/transfac/getTF.cgi?AC=T01049) TGTTT

[HS$GG_22](http://gene-regulation.com/cgi-bin/pub/databases/transfac/getTF.cgi?AC=R02049) 13 (-) 0 100.00 CTAAT

[HS$CATHD_01](http://gene-regulation.com/cgi-bin/pub/databases/transfac/getTF.cgi?AC=R04883) 19 (+) 0 100.00 [ER-alpha](http://gene-regulation.com/cgi-bin/pub/databases/transfac/getTF.cgi?AC=T00261), [Sp1](http://gene-regulation.com/cgi-bin/pub/databases/transfac/getTF.cgi?AC=T00759) GGGCA

[HS$BG_48](http://gene-regulation.com/cgi-bin/pub/databases/transfac/getTF.cgi?AC=R04295) 25 (+) 0 100.00 GGTGG

[CAC-binding protein](http://gene-regulation.com/cgi-bin/pub/databases/transfac/getTF.cgi?AC=T00076)

[HS$BG_52](http://gene-regulation.com/cgi-bin/pub/databases/transfac/getTF.cgi?AC=R03492) 25 (-) 0 100.00 CCACC

[HS$BG_14](http://gene-regulation.com/cgi-bin/pub/databases/transfac/getTF.cgi?AC=R04083) 25 (-) 0 100.00 CCACC

[HS$BG_44](http://gene-regulation.com/cgi-bin/pub/databases/transfac/getTF.cgi?AC=R04291) 25 (-) 0 100.00 CCACC

[CAC-binding protein](http://gene-regulation.com/cgi-bin/pub/databases/transfac/getTF.cgi?AC=T00076)

[HS$ALBU_03](http://gene-regulation.com/cgi-bin/pub/databases/transfac/getTF.cgi?AC=R00079) 27 (+) 0 100.00 [NF-1/L](http://gene-regulation.com/cgi-bin/pub/databases/transfac/getTF.cgi?AC=T00599) TGGCA

[HS$RBP_01](http://gene-regulation.com/cgi-bin/pub/databases/transfac/getTF.cgi?AC=R01322) 27 (+) 0 100.00 [NF-1/L](http://gene-regulation.com/cgi-bin/pub/databases/transfac/getTF.cgi?AC=T00599) TGGCA

[HS$PLOD1_03](http://gene-regulation.com/cgi-bin/pub/databases/transfac/getTF.cgi?AC=R11014) 30 (+) 2 88.89 [PITX2](http://gene-regulation.com/cgi-bin/pub/databases/transfac/getTF.cgi?AC=T02413) CACATGCCTGTAATCCCAGC

[HS$CDH1_01](http://gene-regulation.com/cgi-bin/pub/databases/transfac/getTF.cgi?AC=R11006) 43 (+) 0 100.00 [LUN-1](http://gene-regulation.com/cgi-bin/pub/databases/transfac/getTF.cgi?AC=T04734) TCCCA

[HS$AACS_02](http://gene-regulation.com/cgi-bin/pub/databases/transfac/getTF.cgi?AC=R08505) 55 (+) 0 100.00 [ARP-1](http://gene-regulation.com/cgi-bin/pub/databases/transfac/getTF.cgi?AC=T00045) AGGAGG

[HS$TERT_03](http://gene-regulation.com/cgi-bin/pub/databases/transfac/getTF.cgi?AC=R09485) 57 (-) 1 87.50 [Sp1](http://gene-regulation.com/cgi-bin/pub/databases/transfac/getTF.cgi?AC=T00759) CTCCGCCTC

[HS$EGFR_20](http://gene-regulation.com/cgi-bin/pub/databases/transfac/getTF.cgi?AC=R00393) 66 (-) 0 100.00 TCCTGC

[HS$CDC2_10](http://gene-regulation.com/cgi-bin/pub/databases/transfac/getTF.cgi?AC=R04347) 78 (+) 0 100.00 TTGAA

[HS$CYCA_06](http://gene-regulation.com/cgi-bin/pub/databases/transfac/getTF.cgi?AC=R04360) 78 (+) 0 100.00 TTGAA

[HS$CDC25C_05](http://gene-regulation.com/cgi-bin/pub/databases/transfac/getTF.cgi?AC=R04369) 78 (+) 0 100.00 TTGAA

[HS$ADH3_01](http://gene-regulation.com/cgi-bin/pub/databases/transfac/getTF.cgi?AC=R03017) 79 (+) 0 100.00 [CAR](http://gene-regulation.com/cgi-bin/pub/databases/transfac/getTF.cgi?AC=T02261), [RAR-alpha1](http://gene-regulation.com/cgi-bin/pub/databases/transfac/getTF.cgi?AC=T00719), TGAAC

[RAR-beta](http://gene-regulation.com/cgi-bin/pub/databases/transfac/getTF.cgi?AC=T00721),

[RXR-alpha](http://gene-regulation.com/cgi-bin/pub/databases/transfac/getTF.cgi?AC=T01345)

[HS$GG_12](http://gene-regulation.com/cgi-bin/pub/databases/transfac/getTF.cgi?AC=R00558) 90 (-) 0 100.00 [NF-E](http://gene-regulation.com/cgi-bin/pub/databases/transfac/getTF.cgi?AC=T01214) CTGTC

[HS$INSR_01](http://gene-regulation.com/cgi-bin/pub/databases/transfac/getTF.cgi?AC=R03068) 99 (+) 0 100.00 [C/EBPalpha](http://gene-regulation.com/cgi-bin/pub/databases/transfac/getTF.cgi?AC=T00108) TGCAGTAAG

[HS$CRP_03](http://gene-regulation.com/cgi-bin/pub/databases/transfac/getTF.cgi?AC=R00239) 114 (-) 1 88.89 [C/EBPalpha](http://gene-regulation.com/cgi-bin/pub/databases/transfac/getTF.cgi?AC=T00105), AGTGGCGCAA

[C/EBPbeta](http://gene-regulation.com/cgi-bin/pub/databases/transfac/getTF.cgi?AC=T00581),

[C/EBPdelta](http://gene-regulation.com/cgi-bin/pub/databases/transfac/getTF.cgi?AC=T00109)

[HS$LCK_01](http://gene-regulation.com/cgi-bin/pub/databases/transfac/getTF.cgi?AC=R03518) 133 (-) 1 87.50 [LyF-1](http://gene-regulation.com/cgi-bin/pub/databases/transfac/getTF.cgi?AC=T00479) TCTCCCAGG

[HS$CD11B_01](http://gene-regulation.com/cgi-bin/pub/databases/transfac/getTF.cgi?AC=R04716) 136 (-) 0 100.00 [Sp1](http://gene-regulation.com/cgi-bin/pub/databases/transfac/getTF.cgi?AC=T00759) CGCCC

[HS$GP2B_11](http://gene-regulation.com/cgi-bin/pub/databases/transfac/getTF.cgi?AC=R03304) 139 (-) 0 100.00 [GATA-1](http://gene-regulation.com/cgi-bin/pub/databases/transfac/getTF.cgi?AC=T00306) TTATCG

[HS$EG_08](http://gene-regulation.com/cgi-bin/pub/databases/transfac/getTF.cgi?AC=R03120) 141 (+) 0 100.00 ATAAA

[HS$GFAP_01](http://gene-regulation.com/cgi-bin/pub/databases/transfac/getTF.cgi?AC=R03166) 141 (+) 0 100.00 [TFIID](http://gene-regulation.com/cgi-bin/pub/databases/transfac/getTF.cgi?AC=T00820) ATAAA

[HS$GFAP_02](http://gene-regulation.com/cgi-bin/pub/databases/transfac/getTF.cgi?AC=R03167) 141 (+) 0 100.00 [TBP](http://gene-regulation.com/cgi-bin/pub/databases/transfac/getTF.cgi?AC=T00794) ATAAA

[HS$GG_12](http://gene-regulation.com/cgi-bin/pub/databases/transfac/getTF.cgi?AC=R00558) 152 (+) 0 100.00 [NF-E](http://gene-regulation.com/cgi-bin/pub/databases/transfac/getTF.cgi?AC=T01214) CTGTC

[HS$PTH_04](http://gene-regulation.com/cgi-bin/pub/databases/transfac/getTF.cgi?AC=R11433) 154 (+) 0 100.00 [Ref-1](http://gene-regulation.com/cgi-bin/pub/databases/transfac/getTF.cgi?AC=T04878) GTCTCA
