# Supplementary material for: Both EZH2 and JMJD6 regulate cell cycle genes in breast cancer
Source: BMC Cancer. 2020 Nov 27;20:1159. doi: 10.1186/s12885-020-07531-8 (PMC7694428; doi:10.1186/s12885-020-07531-8)

Fig 3 A- JMJD6 Overexpression western (JMJD6 endogenous and exogenous both visible)

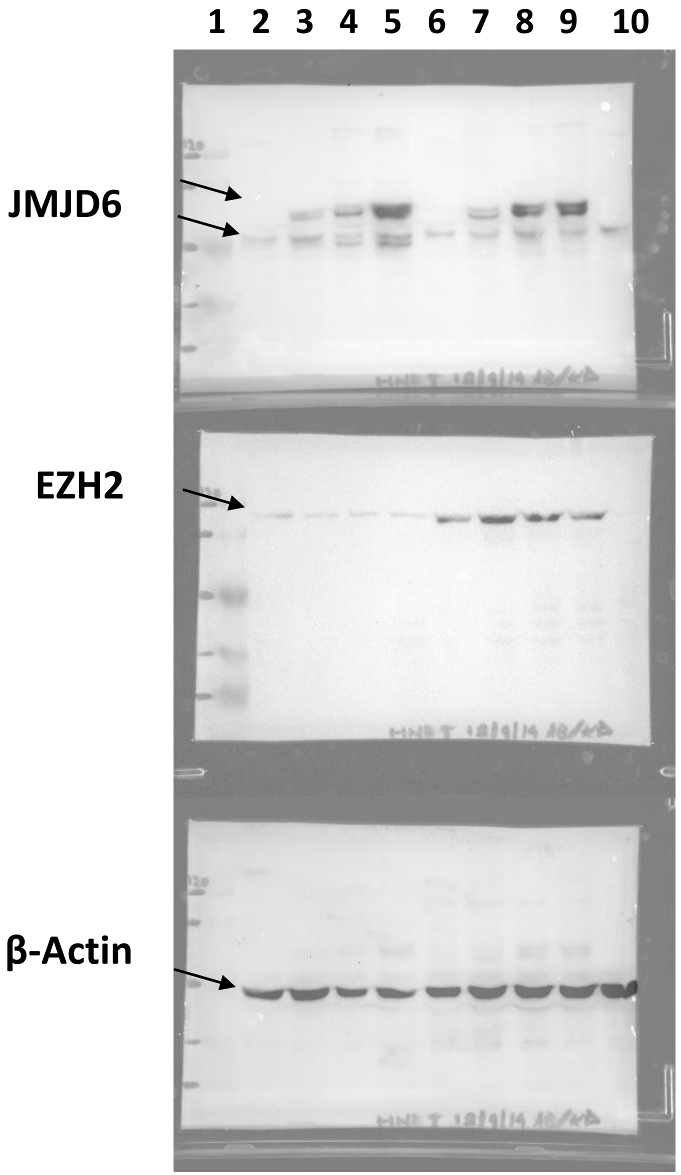

1 – Ladder
2- NA
3- NA
4- NA
5- NA
6- Vec
7- J1-C1
8- J1-C5
9- J1-C6
10-NA

Fig 3 B- siRNA MDA western, JMJD6 oligomers visible

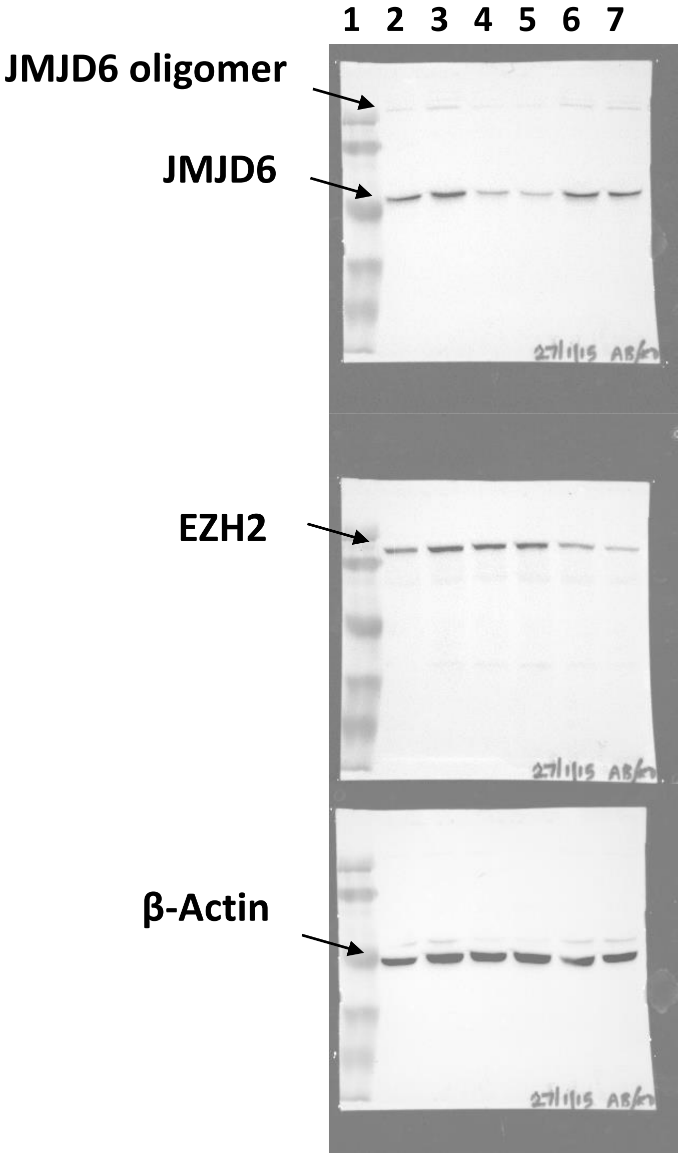

1 – Ladder
2- NA
3- Scr
4- A (JMJD6 siRNA)
5- C (JMJD6 siRNA)
6- E (EZH2 siRNA)
7- F (EZH2 siRNA)

Fig 3 C- EZH2 siRNA HEK western (JMJD6 oligomers visible, EZH2 and actin in same blot, secondary antibody reuse)

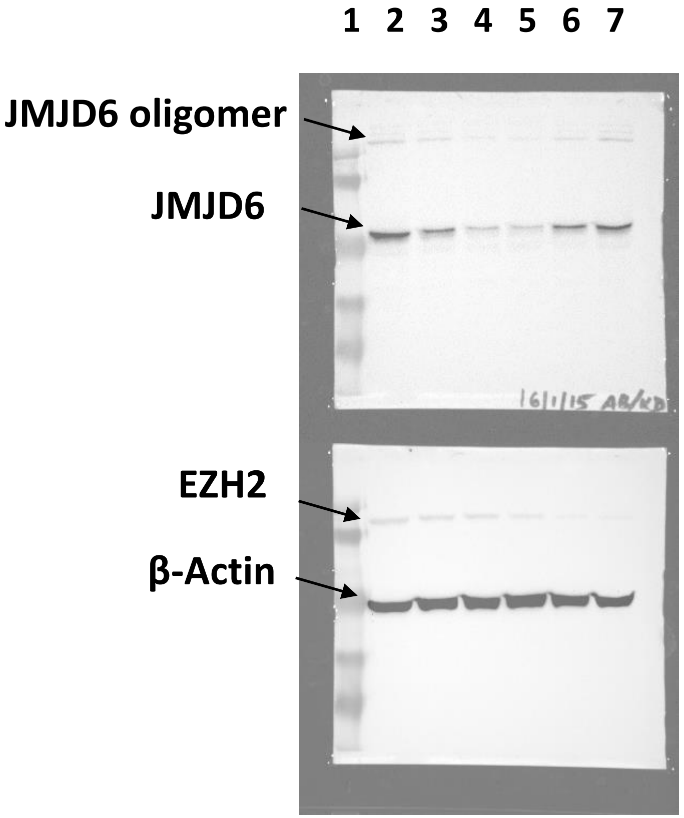

1 – Ladder
2- Scr
3- NA | **Cropped not used in composite figure**
4- NA | **Cropped not used in composite figure**
5- NA | **Cropped not used in composite figure**
6- E (EZH2 siRNA)
7- F (EZH2 siRNA)

Fig 3 C- JMJD6 siRNA HEK western new (EZH2 and JMJD6 in same blot (upper panel), JMJD6 and actin visible in same blot (lower panel), Lanes 5 to 9 belong to another experiment and are cropped out from the composite image in the submitted manuscript

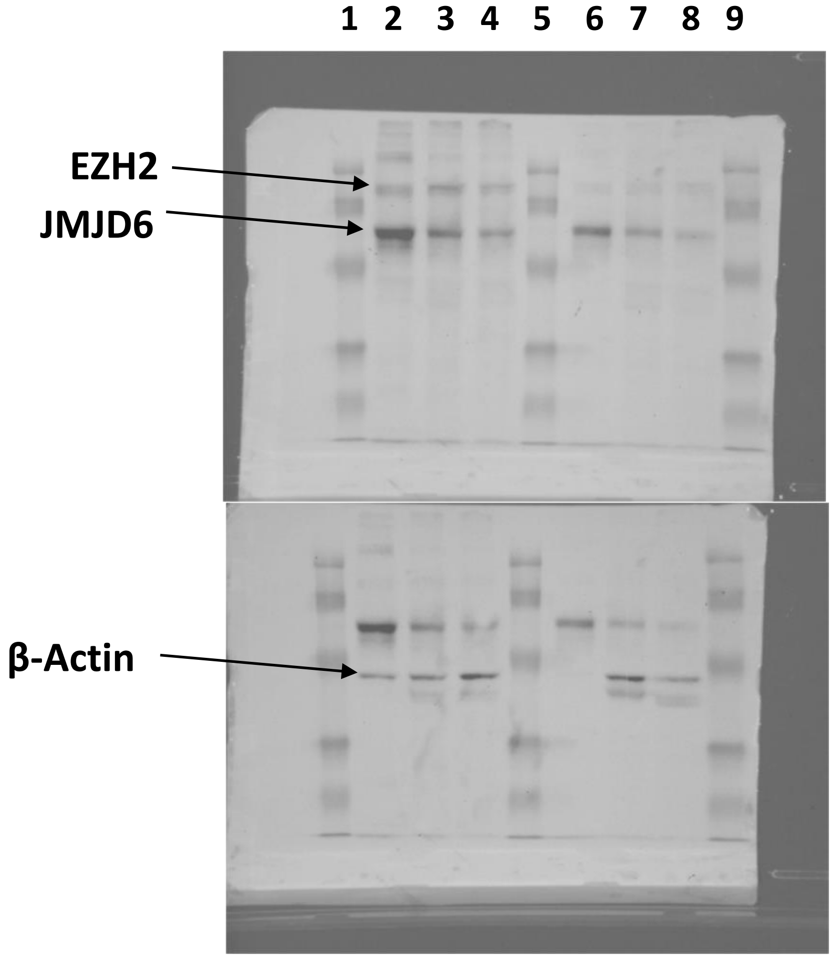

1 – Ladder
2- Scr
3- A (JMJD6 siRNA)
4- C (JMJD6 siRNA)
5- Ladder
6- NA
7- NA
8- NA
9- Ladder

Fig 3 C- JMJD6 siRNA HEK new western

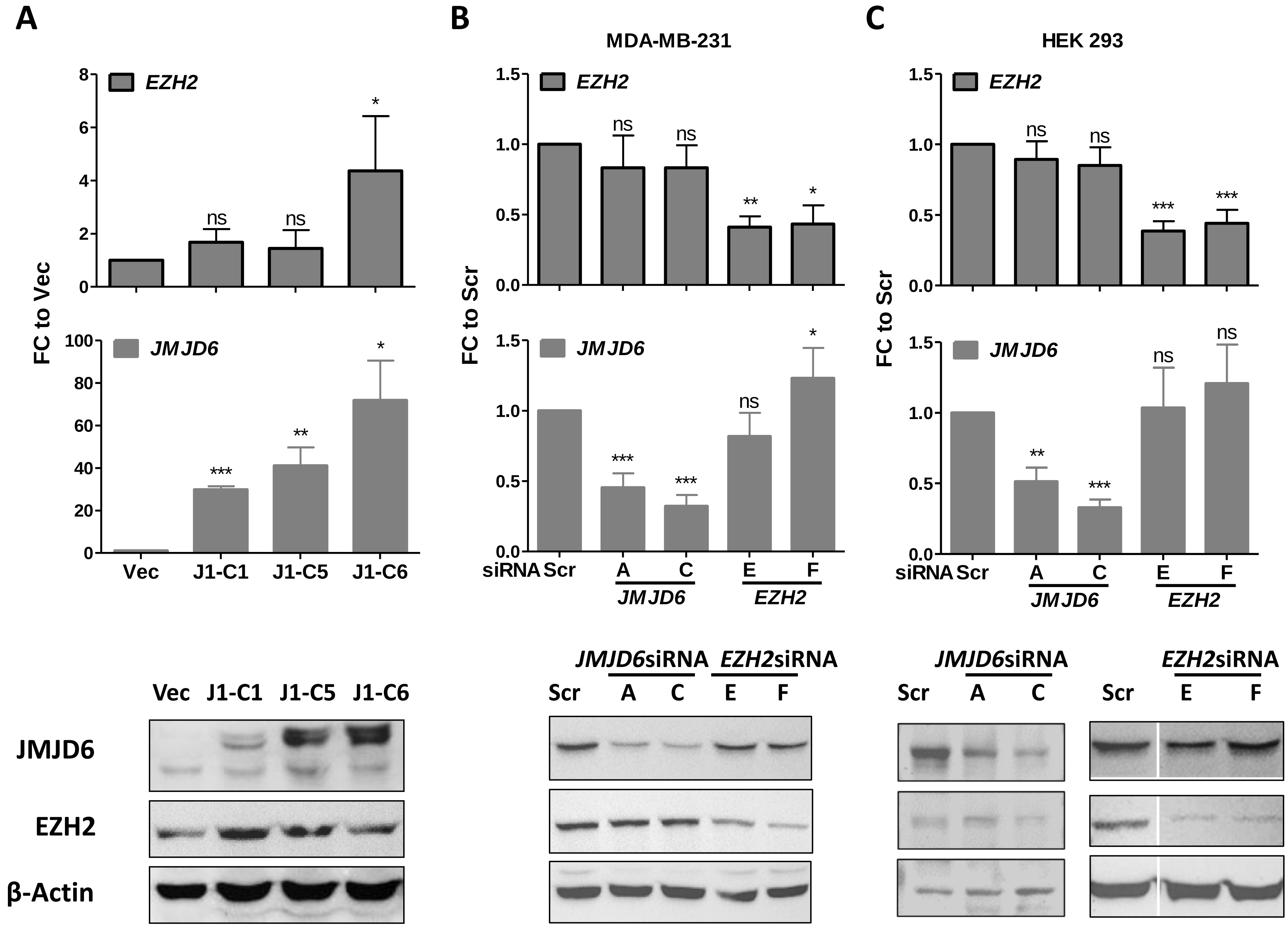

Supplement: Supplementary file 9 — Additional file 9. Original full images of western blots used for generating composite Fig. 3. [file 12885_2020_7531_MOESM9_ESM.pdf]
